# Regulation of formation of volatile compounds of tea (*Camellia sinensis*) leaves by single light wavelength

**DOI:** 10.1038/srep16858

**Published:** 2015-11-16

**Authors:** Xiumin Fu, Yiyong Chen, Xin Mei, Tsuyoshi Katsuno, Eiji Kobayashi, Fang Dong, Naoharu Watanabe, Ziyin Yang

**Affiliations:** 1 Key Laboratory of South China Agricultural Plant Molecular Analysis and Genetic Improvement, South China Botanical Garden, Chinese Academy of Sciences, Xingke Road 723, Tianhe District, Guangzhou 510650, China; 2 Guangdong Provincial Key Laboratory of Applied Botany, South China Botanical Garden, Chinese Academy of Sciences, Xingke Road 723, Tianhe District, Guangzhou 510650, China; 3 University of Chinese Academy of Sciences, No. 19A Yuquan Road, Beijing 100049, China; 4 Tea Research Center, Shizuoka Prefectural Research Institute of Agriculture and Forestry 1706-11 Kurasawa, Kikugawa 439-0002, Japan; 5 Guangdong Food and Drug Vocational College, Longdongbei Road 321, Tianhe District, Guangzhou 510520, China; 6 Graduate School of Science and Technology, Shizuoka University, 3-5-1 Johoku, Naka-ku, Hamamatsu 432-8561, Japan

## Abstract

Regulation of plant growth and development by light wavelength has been extensively studied. Less attention has been paid to effect of light wavelength on formation of plant metabolites. The objective of this study was to investigate whether formation of volatiles in preharvest and postharvest tea (*Camellia sinensis*) leaves can be regulated by light wavelength. In the present study, in contrast to the natural light or dark treatment, blue light (470 nm) and red light (660 nm) significantly increased most endogenous volatiles including volatile fatty acid derivatives (VFADs), volatile phenylpropanoids/benzenoids (VPBs), and volatile terpenes (VTs) in the preharvest tea leaves. Furthermore, blue and red lights significantly up-regulated the expression levels of *9/13-lipoxygenases* involved in VFADs formation, *phenylalanine ammonialyase* involved in VPBs formation, and *terpene synthases* involved in VTs formation. Single light wavelength had less remarkable influences on formation of volatiles in the postharvest leaves compared with the preharvest leaves. These results suggest that blue and red lights can be promising technology for remodeling the aroma of preharvest tea leaves. Furthermore, our study provided evidence that light wavelength can activate the expression of key genes involved in formation of plant volatiles for the first time.

Light is one of the most important environmental factors, acting on plants not only as an essential energy source, but also as an important source of external signal, influencing their growth and development. As plants are empowered with an array of photoreceptors including the blue and UV-A light absorbing cryptochromes, phototropins, the red and far-red absorbing phytochromes, and other implied photoreceptors absorbing in UV-A and green regions^1^, plants not only detect the presence of light, but also its direction, intensity, and wavelength (color). Spectral changes of illumination evoke morphogenetic and photosynthetic responses of plants, and then impact biomass accumulation, stem length, plant primary and secondary metabolism^2^,^3^. Although little is known about regulation of biosynthesis of volatile compounds in plants by light, it is reported that light reflected from red mulch can improve concentrations of aroma compounds in strawberries^4^; volatile profiles of postharvest petunia flowers, tomato, strawberry, and blue-berry can be manipulated with specific light treatments^5^; aroma content of fresh basil leaves can be affected by light reflected from colored mulches^6^. These literatures show some evidences that light has potential to regulate the production of volatile molecules in *planta*.

Tea (*Camellia sinensis*) is one of the important non-alcoholic beverage crops in the world. Aroma (volatile compound) is one of the main sensory properties which are decisive in selection, acceptance, and ingestion of tea. Many attempts have been done to improve or modify the volatiles of tea leaves either by treating leaves during growth of the plants (raw materials), or by postharvest treatment of leaves during the tea manufacturing process^7^. Shade management is a common approach to regulate light conditions during the growth of tea plants^8^. For example, Gyokuro and Tencha, known as the finest teas in Japan, are produced from leaves grown under ceiling-shelf covering. Our previous study indicated that tea leaves kept in darkness by shading treatment for 3 weeks developed etiolated leaves with significantly increased levels of volatiles, especially volatile phenylpropanoids/benzenoids (VPBs)^9^. On the other hand, in response to prolonged shading, tea plants may accelerate their reproductive development, potentially leading to decreases in biomass^10^. To avoid the reduction of biomass, specific light wavelength could be considered for regulation of preharvest tea volatiles. Light emitting diodes (LED) as artificial light source for regulation of plant growth show several advantages including high energy-conversion efficiency, wavelength specificity, light intensity/quality adjustability, and low thermal energy output^3^, and LED irradiations have been successfully applied to cultivations of some crops such as pepper^11^, lettuce^12^, wheat^13^, spinach^14^, banana^15^, pea^3^, and grape^16^. In the present study, we attempted to investigate influences of LED irradiation-based light wavelength on volatile profiles, and their related metabolite levels and gene expressions of preharvest leaves of tea plants. In addition, we also investigated effect of light wavelength on formation of volatiles in postharvest tea leaves and evaluated if LED could be employed to tea manufacturing process to improve tea aroma quality.

## Results and Discussion

### Blue and red lights significantly increased most endogenous volatiles in the preharvest tea leaves

**Figure 1:**
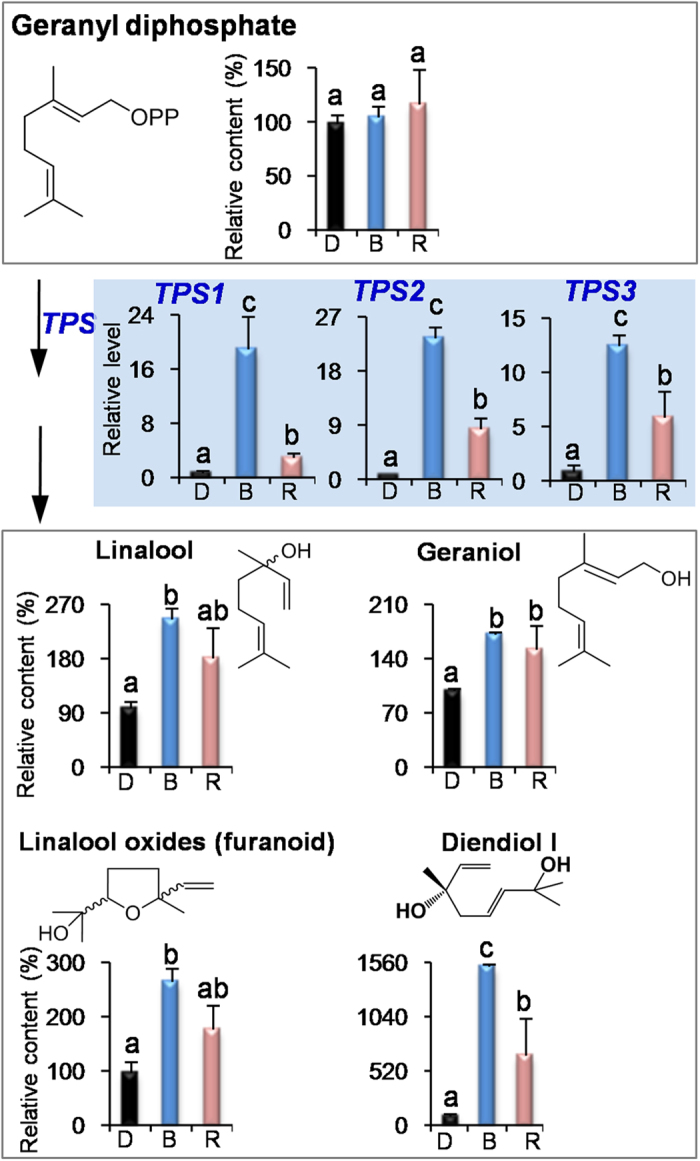
Effects of blue light and red light on the pathway leading from geranyl diphosphate to volatile terpenes in preharvest tea leaves. D, dark treatment. B, blue light treatment. R, red light treatment. *TPS, terpene synthases.* All treatments on preharvest tea leaves were carried out for 3 days. The relative content (%) of metabolites was calculated based on the dark treatment (100%) as a control. The relative level of genes was calculated based on the dark treatment (1) as a control. Different means with different letters are significantly different from each other (*p* ≤ 0.05).

**Figure 2:**
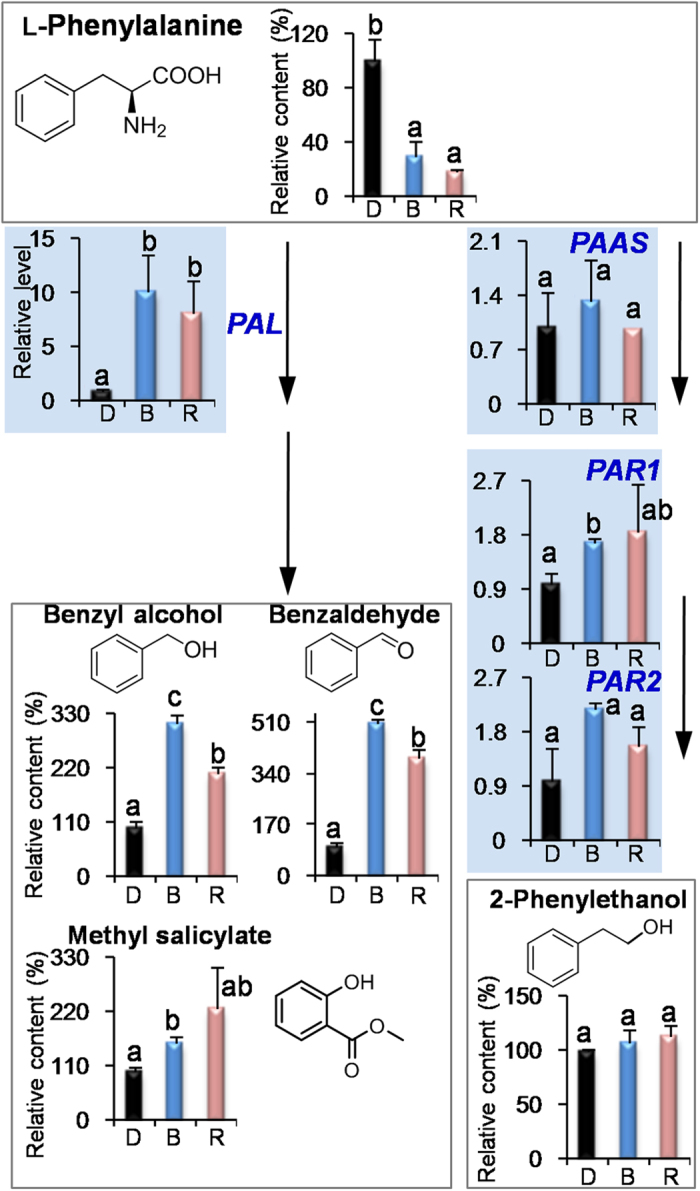
Effects of blue light and red light on the pathway leading from l-phenylalanine to volatile phenylpropanoids/benzenoids in preharvest tea leaves. D, dark treatment. B, blue light treatment. R, red light treatment. *PAL*, *phenylalanine ammonialyase*. *PAAS, phenylacetaldehyde synthase*. *PAR, phenylacetaldehyde reductases*. All treatments on preharvest tea leaves were carried out for 3 days. The relative content (%) of metabolites was calculated based on the dark treatment (100%) as a control. The relative level of genes was calculated based on the dark treatment (1) as a control. Different means with different letters are significantly different from each other (*p* ≤ 0.05).

**Figure 3:**
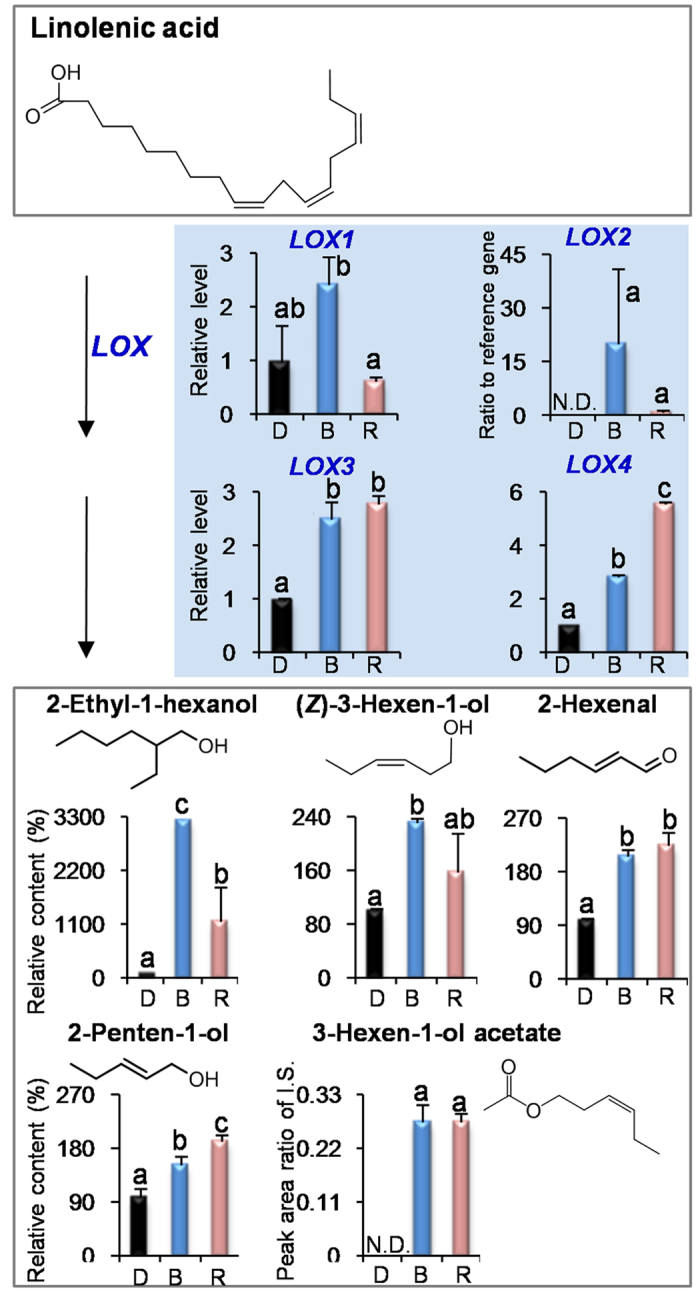
Effects of blue light and red light on the pathway leading from linolenic acid to volatile fatty acids in preharvest tea leaves. D, dark treatment. B, blue light treatment. R, red light treatment. N.D., not detected. I.S., internal standard. *LOX*, *9/13-lipoxygenases*. All treatments on preharvest tea leaves were carried out for 3 days. The relative content (%) of metabolites was calculated based on the dark treatment (100%) as a control. The relative level of genes was calculated based on the dark treatment (1) as a control. Different means with different letters are significantly different from each other (*p* ≤ 0.05).

The preharvest tea leaves contained the three classes of major volatiles including volatile fatty acid derivatives (VFADs), VPBs, and volatile terpenes (VTs). In our preliminary experiment, LED was applied to treat preharvest tea leaves from the 1^st^ leaf stage to the 4^th^ leaf stage (14 days), and the results showed that in contrast to dark treatment as a control, blue light and red light increased the contents of VFADs and VPBs, whereas near-infrared light had less effect on tea volatiles. In addition, VTs decreased or were not affected by the long term (14 days) LED treatments (Fig. S1, Supplementary information). In the present study, 3 days of blue light and red light treatments on the 4^th^ leaf stage of preharvest tea leaves increased most endogenous volatiles including VFADs, VPBs, and VTs compared with dark treatment (Figs 1, 2, 3).

The effect of light wavelength is not limited to growing and development properties of plants. Recent studies indicated that light wavelength can affect the levels of pigments and metabolites such as chlorophylls, carotenoids, phenolic compounds, anthocyanins, ascorbic acid, amino acids, organic acids, fatty acids, sugars, and nitrates^17^,^18^,^19^,^20^,^21^,^22^,^23^. Besides these non-volatile compounds, volatile profiles can also be modulated by specific light wavelengths, which were mostly investigated in flowers and fruits^5^,^24^. In the present study, blue and red lights promoted accumulations of most endogenous volatiles in the tea leaves (Figs 1, 2, 3), providing an evidence of modulation of leaf volatiles by light wavelength. In fruits of tomato, strawberry, and blueberry, the light wavelength did not significantly affect or increase the accumulation of VFADs compared with the dark treatment^5^. In contrast, VFADs in tea leaves were significantly increased by blue and red lights (Fig. 3). In petunia flowers, red and far-red lights had more effects on VPBs and blue light had little or no effect on them^5^, whereas VPBs in tea leaves had more response to blue and red lights (Fig. 2). These differentiations suggested that even the same class of volatiles in different plant species and tissues may have different response patterns to light wavelength.

In our previous report^9^, shading treatment (dark) increased endogenous VFADs and VPBs in contrast to the non-shading treatment. However, biomass of tea leaves often reduced under dark condition. Application of blue and red lights did not significantly affect the biomass of growing tea leaves in contrast to the natural light/non-shading treatment (Fig. S2). Furthermore, blue and red lights increased most tea volatiles (Figs 1, 2, 3) even compared with the dark treatment. These suggested that single wavelength application had more advantages for ensuring yield and improving quality than the shading treatment (dark). Interestingly, we found that VPBs and VFADs were increased either under long-term (14 days) LED treatment (Fig. S1) or under short-term (3 days) supplement LED treatment (Figs 2 and 3), indicating that the both classes of volatiles were easily modulated by blue light and red light regardless of the leaf development stage. In contrast, VTs level was up-regulated by blue and red lights only under the short-term (3 days) supplement LED treatment (Fig. S1 and Fig. 1).

The three classes of volatiles, VFADs, VPBs, and VTs, contribute to different odour characters of teas. Most VFADs, such as hexanol and (*Z*)-3-hexen-1-ol, show green/grassy note, whereas VPBs (for example, benzyl alcohol and 2-phenylethanol) and VTs (for example, linalool and geraniol) show floral odour^7^. In the present study, blue and red lights can achieve the enhancement of contents of the three classes of volatiles. As the odour characters of teas are affected by several factors including concentrations of volatiles, ratios of different volatiles, and odor threshold, and different people have different preferences, further works on dual regulation of concentration and ratio of volatiles are required to make desired tea aroma that meeting different demands.

### Blue and red lights activated the expressions of key genes involved in formation of volatiles in preharvest tea leaves

To further find out how blue and red lights increase the volatiles in the preharvest tea leaves, we investigated the effects of blue and red lights on the pathways of VTs, VPBs, and VFADs including key precursors and genes. At present, analytical methodology of geranyl diphosphate (GDP) in tea leaves is unavailable. Firstly we established an acid hydrolysis of GDP to determine GDP amount by measuring the amounts of volatiles from the acidic hydrolysis of GDP. Linalool was positively recognized as a major volatile product of the acidic hydrolysis of GDP (Fig. S3A). Furthermore, a good linear relationship was established between GDP amount and its acidic hydrolyzed product (linalool) content, although in the acidic hydrolysis system, GDP was not completely (or 100%) transformed to linalool (Fig. S3B). In contrast to the dark treatment, blue and red lights did not increase the level of GDP that leading to the formation of VTs (Fig. 1), and decreased the level of l-phenylalanine (l-Phe), which is a key precursor of VPBs (Fig. 2). α-Linolenic acid was not detected in tea leaves, which may be due to that its content was below detection limit. However, linoleic acid was detected and its content was not increased by the light wavelength (Fig. S4). Interestingly, blue and red lights significantly up-regulated the expression levels of *terpene synthase* (*TPS*) involved in the formation of VTs, *phenylalanine ammonialyase* (*PAL*) involved in the formation of VPBs, and *9/13-lipoxygenase* (*LOX*) involved in the formation of VFADs (Figs 1, 2, 3). In addition, in contrast to the natural light treatment, blue light and red light also significantly increased the expression levels of these genes (Fig. S5). These suggest that blue and red lights can activate these gene expressions.

Although recent studies have demonstrated that single wavelength can affect the metabolite levels of plants, the information on detailed mechanism of regulation by single wavelength is still very limited. The present study showed evidence that blue and red lights can activate the expressions of key genes such as *TPS*, *PAL*, and *LOX* involved in formation of volatiles in preharvest tea leaves, which is the first report on interaction between single light and expressions of genes involved in plant volatiles. *LOX* and *PAL* are located in cytosol, whereas *TPS* is located in plastid. In addition, some genes that are not affected by the blue and red lights such as *phenylacetaldehyde synthase* (*PAAS*) and *phenylacetaldehyde reductases* (*PAR*) are located in cytosol^25^,^26^. Further investigation on interaction between activations of phytochrome and cryptochromes (or possibly other light sensors) and up-regulation of these gene expressions may explain how blue and red lights activate these gene expressions.

The largest class of plant volatiles is derived from isoprenoid pathways. The isoprenoid pathways include cytosolic mevalonate and the plastidic methylerythritol phosphate pathways. Diverse hemi-, mono-, sesqui-, and diterpene plant volatiles are derived from dimethylallyl pyrophosphate, GDP, farnesyl pyrophosphate, and geranylgeranyl diphosphate, respectively^25^. In fresh tea leaves, most volatile terpenes are mono-terpenes that are derived from GDP^7^. Analytical result of GDP amounts of tea leaves showed that blue and red lights did not significantly affect the amount of GDP in contrast to the dark treatment (Fig. 1), suggesting the GDP content was not influenced by light wavelength. As GDP can be converted to volatile mono-terpenes under the action of *TPS*, we investigated the effects of blue and red lights on the *TPS1*, *TPS2*, and *TPS3*, which are possibly involved in formation of linalool and geraniol in tea leaves^27^. Furthermore, the *TPS1* and *TPS3* recombinant proteins produced in *Escherichia coli* were demonstrated to exhibit the activity of transformation from GDP to linalool (Fig. S6A,B). The three *TPS* can be up-regulated by blue and red lights.

The second most ubiquitous class of plant volatiles consists of VPBs containing an aromatic ring. Most volatiles from this class are derived from shikimate *via* l-Phe. The first committed step in the biosynthesis of many VPBs is the deamination of l-Phe to *trans*-cinnamic acid (CA) by the enzyme PAL^25^, whereas the biosynthesis of other VPBs- related compounds such as 2-phenylethanol, does not occur *via* CA and competes with PAL for l-Phe utilization, and is formed under the actions of PAAS and PAR^28^,^29^. In the present study, l-Phe amount of tea leaves exposed to blue and red lights was much lower than that of dark treatment (Fig. 2). In our previous study, most amino acids including l-Phe were present at higher levels in dark treated leaves in contrast to tea leaves exposed to light condition, which may be due to the degradation of proteins under dark-induced carbohydrate starvation^9^. In contrast, expression level of *PAL* gene was significantly up-regulated by blue and red lights, which may lead to big increase in benzyl alcohol and benzaldehyde (Fig. 2). The PAL was reported to be involved in the pathway from l-Phe to CA in tea leaves^30^ and PAL recombinant protein produced in *E. coli* exhibited the activity of transformation from l-Phe to CA (Fig. S6A,C). Blue and red lights did not affect the amount of 2-phenylethanol.This may be due to that blue and red lights did not increase expression levels of *PAAS* and *PAR* genes, which are likely involved in the formation of 2-phenylethanol in tea leaves (Figs S7 and S8). These results suggested that activation of gene expressions played important role in regulation of formation of tea volatiles by single wavelength.

A third group of plant volatiles is derived by oxidative cleavage and decarboxylation of various fatty acids, resulting in the production of shorter-chain volatiles with aldehyde and ketone moieties. These compounds originate from C18 unsaturated fatty acids (linoleic or linolenic acids), which enter the “lipoxygenase pathway”. The first step of this pathway is the dioxygenation of unsaturated fatty acids, catalyzed by LOX. The *LOX2, LOX3*, and *LOX4* gene expressions were up-regulated by blue and red lights, which may result in the increase of VFADs (Fig. 3). We found the four *LOX* in tea leaves (Fig. S9). The *LOX1* identified in tea leaves is proposed as a wounding stress-response gene^31^. The other three genes are proposed to likely be light wavelength-response genes. As genetic transformation system of tea is not successfully established yet, it is not easy to characterize the functions of enzymes involved in biosynthesis of plant metabolites, especially in tea plants. Further studies on *in vitro* and *in vivo* functional characterization of the enzymes involved in the biosynthesis of volatile compounds are helpful for completely understanding the link between transcript and metabolite level.

### Single light wavelength had less significant influences on the formation of volatiles in the postharvest leaves compared with the preharvest leaves

**Figure 4:**
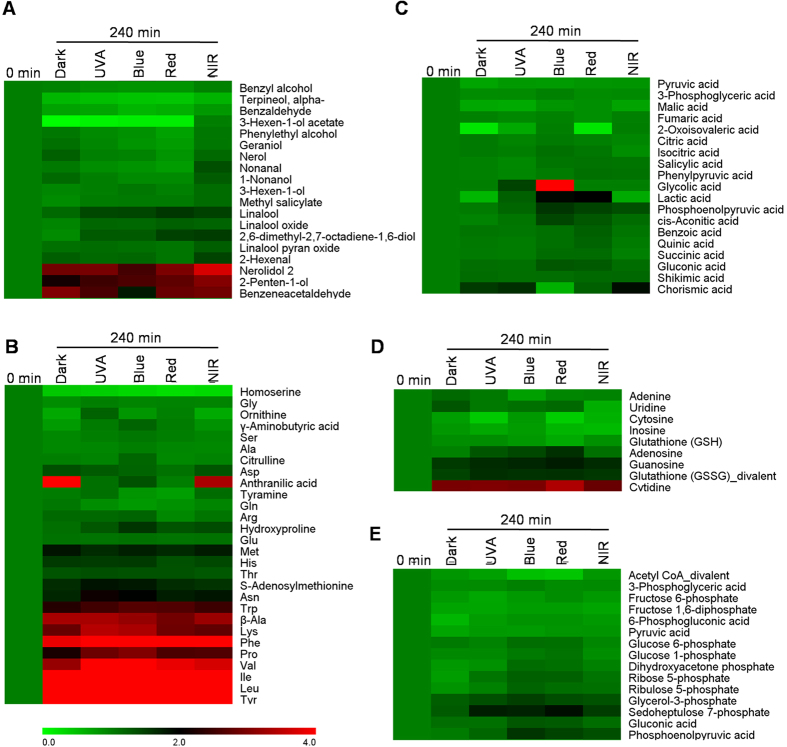
Comparison of metabolites in 0 min and 240 min treatments of dark, UVA, blue light, red light, and near-infrared (NIR) on postharvest tea leaves. (**A**) volatiles. (**B**) amino acids. (**C**) organic acids. (**D**) alkaloid. (**E**) metabolites involved in glycolysis and pentose pathways. The 0 min treatment was normalized as 1. The data were calculated based on mean values (n = 3). The data for making this figures is shown in Tables S3 and S4 (Supplementary information). Full names of metabolites are given in Table S5.

**Figure 5:**
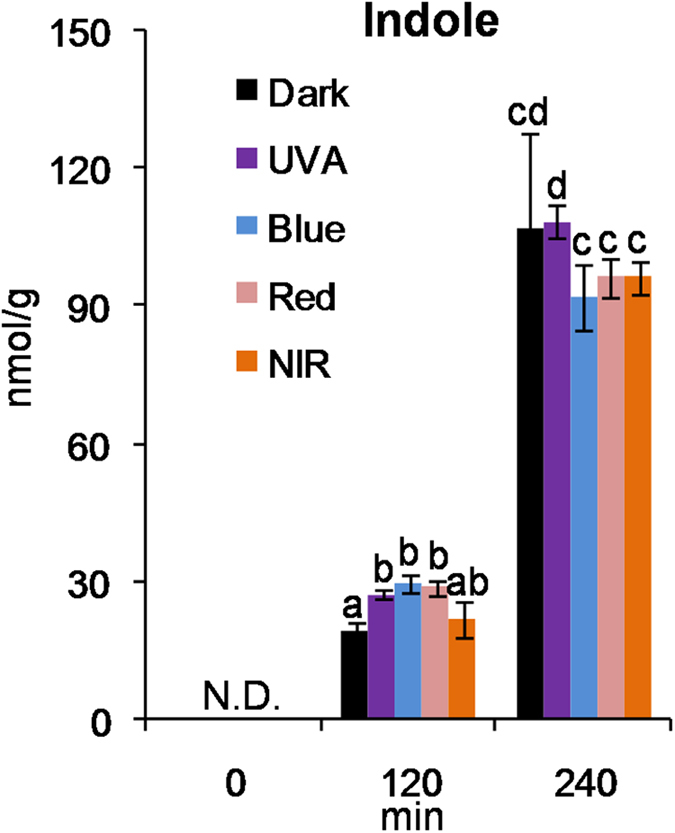
Comparison of indole in 0 min, 120 min, and 240 min treatments of dark, UVA, blue light, red light, and near-infrared (NIR) on postharvest tea leaves. N.D., not detected. Different means with different letters are significantly different from each other (*p* ≤ 0.05).

As tea volatiles vary with the manufacturing process, we also investigated effects of single light wavelength on formation of volatiles in the postharvest tea leaves. In contrast to the dark condition, single light wavelength did not show significant effect on tea volatiles either after treatments of 120 min (Fig. S10) or 240 min (Fig. 4A). The non-volatile metabolites including amino acids, alkaloid, organic acids, and metabolites involved in glycolysis and pentose pathways were also not affected by single light wavelength in contrast to the dark condition (Fig. 4B–E). However, some amino acids (Fig. 4B) and indole (Fig. 5) increased under all the 240 min-treatments in contrast to the 0 min-treatment, suggesting that these metabolites may be majorly regulated by wounding stress induced by picking.

It is generally accepted that manufacturing process has remarkable effects of formation of tea aroma. For example, fermented teas including oolong tea and black tea have more aroma characters than non-fermented tea, green tea. During the oolong tea manufacturing process, tea leaves are exposed to various stresses, such as plucking (wounding), solar withering (drought, heat, and UV/light radiation), indoor withering (drought), and turn over (wounding)^32^. During the black tea manufacturing process, tea leaves are exposed to cell disruption and wounding stresses. Most reports tend to indicate that wounding stress, cell disruption, and temperature have more obvious effects on tea volatiles within the manufacturing process^33^,^34^,^35^,^36^. In the present study, single light wavelength had less remarkable influence on the formation of volatiles in the postharvest leaves compared with the preharvest leaves, which may be due to the relatively short time application of single light wavelength on postharvest leaves. This may also suggest that metabolite levels of tea leaves require a relative long time to respond to single light wavelength. As manufacturing process of postharvest tea leaves has a certain time limitation, it is not feasible to apply a long time of single light wavelength treatment to the postharvest tea leaves.

In this study, LED was employed to improve tea aroma. In contrast to shading treatment (dark), it did not significantly affect the biomass of tea leaves and further improve tea aroma. Blue light and red light were selected as promising technology for remodeling the aroma of preharvest tea leaves. Furthermore, our study provided evidence that blue light and red light can activate the expressions of key genes involved in formation of plant volatiles for the first time. This information will advance our understanding of the effects of environmental factors such as light on metabolites in tea leaves, and present an opportunity to manipulate aroma in raw materials of tea without adding transgenes, treating with hormones or affecting plant nutrition.

## Methods

### Treatment of LED on the preharvest tea leaves

Tea leaves (*C. sinensis* var. Jinxuan; one bud and three leaves stage; grown in April) were exposed to irradiations of blue light (470 nm, 70–80 μmol/m^2^/s) and red light (660 nm, 70–80 μmol/m^2^/s) (Shenzhen FHT Electronics Technology Co., Ltd.) for 3 days. The dark treatment was used as a control. The light conditions of blue light, red light, and natural light are indicated in Fig. S11. The tea leaves grown in natural light was used as another reference. All the treatments were performed under a constant temperature of 22 °C.

### Treatment of LED on the postharvest tea leaves

In postharvest experiments, two hundred gramme of freshly picked tea leaves (the first crop) were exposed to irradiations (30 cm × 30 cm) of ultraviolet-A, blue light (470 nm, 70–80 μmol/m^2^/s), red light (660 nm, 70–80 μmol/m^2^/s), near-infrared (730 nm, below 0.1 μmol/m^2^/s), and dark (control) for 0, 120, and 240 min, respectively.

### Analysis of endogenous volatiles of preharvest and postharvest tea leaves by gas chromatography-mass spectrometry (GC-MS)

To minimize the effect of enzyme activation and determine the “natural” volatile compounds in fresh tea leaves, direct organic solvent extraction was applied to extraction of volatiles^9^,^37^.

For the preharvest tea leaves, 400 mg of the tea leaves fine powder crushed by liquid nitrogen was extracted with 1.5 mL of dichloromethane containing ethyl *n*-decanoate as an internal standard overnight under dark condition, centrifugated (2,000 *g*, 4 °C, 5 min), and then dried over anhydrous sodium sulfate. The resulting solution was concentrated to 100 μL using N_2_, and then subjected to a GC-MS QP2010 SE (Shimadzu Corporation, Japan) equipped with a SUPELCOWAX^TM^ 10 column (30 m × 0.25 mm × 0.25 μm, Supelco Inc., Bellefonte, PA, USA). The injector temperature was 240 °C, splitless mode was used with a splitless time of 1 min, and helium was the carrier gas with a velocity 1.0 mL/min. The GC temperatures were 60 °C for 3 min, ramp of 4 °C/min to 180 °C, followed by 20 °C/min to 240 °C, then 240 °C for 15 min. MS was performed in full scan mode (mass range *m/z* 40–200).

For the postharvest tea leaves, one gramme of tea leaves fine powder were extracted with 2.5 mL of diethyl ether containing ethyl *n*-decanoate as an internal standard overnight under dark condition, and then dried over anhydrous sodium sulfate. One μL of the resulting solution was subjected to GC-MS (Agilent 7890 gas chromatograph with 5975 mass spectrometry), equipped with an InertCap PureWAX, 60 m × 0.25 mm I.D., and 0.25 μm film thickness (GL Sciences Inc., Japan). Helium was used as a carrier gas at velocity 2.05 mL/ min. The temperature of injector was 240 °C. The GC temperatures were 40 °C for 1 min, ramps of 5 °C/min to 250 °C and held for 15 min. The MS analyses were carried out in a full scan mode with scan range 20–280 amu. Electron impact ionisation at energy 70 eV was used for every measurement. The ion source and quadrupole analyzer temperatures were fixed at 230 °C and 150 °C, respectively.

### Analysis of amino acids in preharvest tea leaves by amino acid analyzer

Plant tissues (100 mg fresh weight, finely powdered) were extracted with 0.5 mL cold methanol by vortexing for 2 min followed by ultrasonic extraction in ice cold water for 15 min. The extracts were mixed with 0.5 mL chloroform and 0.2 mL cold water for phase separation, and the resulting upper layer was dried, and was added with 1 mL of 5% sulfosalicylic acid, and stood for 1 h. After centrifuging at 5000 × *g* for 10 min, the supernatant was filtered through 0.45 μm membrane, and subjected to an amino acid analyzer. A Sykam S433D Physiological Li C4 system (SYKAM GmbH, Eresing, Germany) equipped with quaternary pump, column oven, refrigerated auto sampler, and UV-Vis detector was used. The Physiological Li C4 system was coupled with S4300 post-column derivatization system. The experiments were performed on a high efficiency sodium cation-exchange Pickering Laboratories column (4.0 × 150 mm). The Sykam S433D Physiological Li C4 system was operated using a mobile phase consisting of lithium citrate pH 2.9, pH 4.2, pH 8.0, and using UV-Vis detection at 570 nm and 440 nm. The flow-rate of the mobile phase was 0.45 mL/min, and the flow-rate of the derivatizating reagent was 0.25 mL/min. The column temperature was set at 38 °C, and the post column reaction equipment was kept at 130 °C temperature. The temperature of the auto-sampler was kept at 5 °C, and the injection volume was 50 μL for both standard and samples.

### Analysis of linolenic acid and linoleic acid contents in preharvest tea leaves

The total fatty acid profile was determined according to Zhang *et al.*^38^. Briefly, 0.5 g of frozen powdered tea leaves was used to extract total lipids by gentle shaking in a mixture of chloroform/methanol/water (1: 2: 0.8, v/v/v). After centrifugation at 5000 × *g* for 15 min at 4  °C, the upper phase was extracted with 0.8 mL chloroform and NaCl (0.76%, w/v). The residue was dissolved in 2 mL hexane and stored at −20 °C for further analysis. Total fatty acids were transformed into their corresponding FAME by the addition of 2 mL of H_2_SO_4_ (2.5% in methanol, v/v), and a known amount of heptadecanoic acid (C17:0) was added as internal standard. A GC (Agilent 6890N) equipped with a ultra 2 column (0.20 mm × 25 m, 0.33 μm, Agilent19091B-102) was used for fatty acid analysis. Injection was in the split ratio (100:1) mode at 250 °C. The initial oven temperature was 170 °C, and it increased to 260 °C at 5 °C min^−1^ and then to 310 °C at 40 °C min^−1^. Helium was the carrier gas at a flow rate of 1.0 mL/min. Identification of compounds was confirmed by comparing them with authenticated reference standards (Sigma).

### Analysis of GDP contents in preharvest tea leaves

Based on the reference^39^, we optimized a method for determination of GDP in tea leaves. One hundred mg of fresh tea leaves fine powder were extracted with 1 mL buffer (50 mM Tris/HCl pH 8.0, 20 mM DTT, 20 mM MgCl_2_, and 5% glycerin) containing 10 mM sodium molybdate as phosphatase inhibitor by vortexing for 2 min followed by ultrasonic extraction in ice cold water for 10 min, and centrifuged (16,400 *g*, 4°C, 15 min). The supernatant (160 μL) was extracted with hexane (200 μL, 4 times) to remove free volatiles. The 40 μL above buffer was added and the aqueous fraction was incubated at 37 °C for 30 min with 100 μL of 1 M H_2_SO_4_. The reaction solution was cooled down for 3 min on ice and then the reaction was stopped by adding 100 μL of 4.0 M NaOH. The resultant mixture was extracted with 400 μL of hexane containing 0.5 nmol of ethyl decanoate as internal standard. The upper layer was dried over Na_2_SO_4_ and analyzed by GC-MS QP2010 SE (Shimadzu Corporation, Japan) equipped with a SUPELCOWAX^TM^ 10 column (30 m × 0.25 mm × 0.25 μm, Supelco Inc., Bellefonte, PA, USA). The injector temperature was 240 °C, splitless mode was used with a splitless time of 1 min, and helium was the carrier gas with a velocity 1.0 mL/min. The GC temperatures were 60 °C for 3 min, ramp of 4 °C/min to 180 °C, followed by 20 °C/min to 240 °C, then 240 °C for 15 min. MS was performed in full scan mode (mass range *m/z* 40–200).

### Simultaneous determination of amino acids and other metabolites in postharvest tea leaves by capillary electrophoresis-time of flight mass spectrometry (CE-TOFMS)

The metabolites were analyzed by CE-TOFMS as described by previous report^9^. Briefly, 100 mg of fresh tea leaves fine powder were extracted with 1,000 μL of methanol containing 25 μM each of the internal standards (methionine sulfone, 2-morpholinoethansulfonic acid, D-camphor-10-sulfonic acid). Afterwards, 1,000 μL of chloroform and 400 μL of Milli-Q water were added, and then vortexed for 30 sec and centrifugated (2,300 *g*, 4 °C, 5 min). The resultant aqueous layer was obtained and ultrafiltrated by centrifugation (9,100 *g*, 4 °C, 3 h) through 5 kDa-cutoff filter. The filtrate was dried in vacuum, dissolved in 50 μL of Milli-Q water containing 100 μM of the internal standards (3-aminopyrrolidine, trimesic acid, and 2-naphtol-3,6-disulfonic acid) and 50 μM of the internal standards (*N,N*-diethyl-2-phenylacetamide, and diazoxid), and then subjected to CE-TOFMS apparatus (Agilent Technologies) equipped with a fused silica capillary (i.d. 50 μm × 80 cm). For analysis of cationic metabolites, cation buffer solution was used as run buffer and rinse buffer. The ESI positive mode was employed with scan range *m/z* 50–1000. CE voltage and MS capillary voltage were set to 27 and 4 kV, respectively. For analysis of anionic metabolites, anion buffer solution was used as run buffer and rinse buffer. The ESI negative mode with scan range *m/z* 50–1000 was used. CE voltage and MS capillary voltage were set to 30 and 3.5 kV, respectively.

### Transcript expression analysis of key genes involved in formation of tea volatiles

Total RNA was extracted from frozen powder using Plant Total RNA Kit (Huayueyang Biotechnology CO., LTD). The first-strand cDNA was synthesized using a PrimeScript RT Reagent Kit with gDNA Eraser (Takara, Japan). The qRT-PCR analysis reactions were performed in a total of 10 μL, including 0.4 μL of each primer (10 μM), 1 μL cDNA, 5 μL SYBR Premix Ex Taq (TliRNaseH Plus) (Takara, Japan) on a RocheLightCycler 480. The PCR programme was initiated with a preliminary step of 30 s at 95 °C, followed by 40 cycles at 95 °C for 5 s and 60 °C for 20 s. A melting curve was generated for each sample at the end of each run to ensure the purity of the amplified products. No-template controls for each primer pair were included in each run. *β-Actin* was used an reference gene^40^,^41^. The *β-actin*, *LOX*, *PAL*, *PAAS*, *PAR*, and *TPS* gene specific primers of qRT-PCR is shown in Table S1. Change in mRNA level of target gene for each treatment was normalized to that of *β-actin*.

### Gene cloning

To amplify the full length open reading frames (ORFs) of *TPS1*, *TPS3*, and *PAL* genes, the primers in Table S2 were used. PrimeSTAR Max DNA Polymerase (Takara) was used to clone these genes, for its highest fidelity and fastest extension rate. The PCR conditions were shown as follows: denaturation at 98 °C for 30 s, followed by 36 cycles of 98 °C for 10 s, 60 °C for 5 s, 72 °C for 1 min, and a final extension at 72 °C for 10 min. The resulting PCR products were purified, added A base by taq polymerase, and then subcloned into the pGEM-T vector (Promega, USA).

### Expression in *E. coli* and SDS-page identification

The full-length ORFs of the above genes were subcloned into pET32a vector (Novagen, Madison, WI, USA) to obtain the expression construct (pET32a/TPS1, pET32a/TPS3, and pET32a/PAL). After verification by sequencing, the expression construct was transformed into *E*. *coli* Rosetta-gami B (DE3) pLysS for recombinant protein expression. A single colony from each transformed *E*. *coli* Rosetta-gami B(DE3) pLysS cell line was incubated overnight at 37 °C in 1 mL LB containing ampicillin, chloramphenicol, and kanamycin at a concentration of 100 μg/mL, 34 μg/mL, and 15 μg/mL, respectively. Inocula were then individually added to 200 mL LB, containing 100 μg/mL ampicillin, 34 μg/mL chloramphenicol, and 15 μg/mL kanamycin and grown at 37 °C to an OD_600_ at 0.6. After adding 0.1 mM isopropyl-beta-d-thiogalactopyranoside (Sigma-Aldrich), the cultures were grown at 20 °C for another16 h to produce recombinant protein. The cells were harvested at 10,000 × *g* for 10 min, then disrupted by sonication for 20 min in a 50 mM NaH_2_PO_4_ (pH 8.0) buffer containing 300 mM NaCl and 10 mM imidazole. After centrifugation at 10,000 × *g* for 20 min, the supernatant was collected and purified by using affinity binding on Ni Sepharose 6 Fast Flow (GE Healthcare, USA) according to the manufacturer’s instruction. The purified protein was passed through a PD-10 (GE Healthcare,USA) desalting column. The protein samples were diluted 1:2 with SDS loading buffer and denatured for 10 min at 100 °C. SDS-PAGE was performed using 10% (w/v) polyacrylamide gels. Unstained standard proteins (Bio-Rad, Munich, Germany) were used for the preparation of a calibration curve for the determination of molecular masses.

### Enzyme assays

The enzyme activity of *TPS1* and *TPS3* recombinant protein produced in *E. coli* was determined as described by the ref. 42, with a modification. The enzyme assay was carried out in 1 mL reaction buffer (pH 7.5 50 mM Tris-HCL, 10 mM MgCl_2_, and 5 mM DTT) containing 50–100 μg purified recombinant protein and 20 μM GDP substrate in a 50 mL glass tube. The mixture was reacted at 30 °C and a solid-phase microextraction (SPME) fiber (2 cm–50/30 μm DVB/CarboxenTM/PDMS Stable FlexTM from Supelco) was used to collection of the volatiles from the enzyme reaction. The collection time of SPME fiber was 30 min. Blanks were carried out using the boiled enzyme or pET32a vector enzyme instead of the fresh recombinant protein. The SPME was subjected to the GC-MS analysis described as above.

The enzyme activity of PAL recombinant protein produced in *E. coli* was determined as described by the ref. 43. The reaction solution was 1.5 mL 50 mM Tris-HCl (pH 8.8) containing 10 μL of 0.04 mM l-Phe and 0.5–2 μg of purified PAL recombinant protein. The enzyme assay was performed at 30 °C. The reaction product CA was determined using UV absorption measurement. The reaction solution was measured at 290 nm every 10 s for 60 min. The PAL was assayed from the increase in absorbance at 290 nm. The control was carried out using the pET32a vector enzyme instead of the fresh recombinant protein.

## Additional Information

**How to cite this article**: Fu, X. *et al.* Regulation of formation of volatile compounds of tea (*Camellia sinensis*) leaves by single light wavelength. *Sci. Rep.* **5**, 16858; doi: 10.1038/srep16858 (2015).

## Supplementary Material

Supplementary Information
