# Supplementary material for: Regulation of formation of volatile compounds of tea (Camellia sinensis) leaves by single light wavelength
Source: Sci Rep. 2015 Nov 16;5:16858. doi: 10.1038/srep16858 (PMC4645219; doi:10.1038/srep16858)
Supplement: Supplementary Information [file srep16858-s1.pdf]

Title: Regulation of formation of volatile compounds of tea (*Camellia sinensis*) leaves by single light wavelength

Authors: Xiumin Fu [1, 2, +], Yiyong Chen [1, 3, +], Xin Mei [1, 2], Tsuyoshi Katsuno [4], Eiji Kobayashi [4], Fang Dong [5], Naoharu Watanabe [6], and Ziyin Yang [1, 2, 3, *]

Affiliations:

[1] *Key Laboratory of South China Agricultural Plant Molecular Analysis and Genetic Improvement, South China Botanical Garden, Chinese Academy of Sciences, Xingke Road 723, Tianhe District, Guangzhou 510650, China*

[2] *Guangdong Provincial Key Laboratory of Applied Botany, South China Botanical Garden, Chinese Academy of Sciences, Xingke Road 723, Tianhe District, Guangzhou 510650, China*

[3] *University of Chinese Academy of Sciences, No.19A Yuquan Road, Beijing 100049, China*

[4] *Tea Research Center, Shizuoka Prefectural Research Institute of Agriculture and Forestry 1706-11 Kurasawa, Kikugawa 439-0002, Japan*

[5] *Guangdong Food and Drug Vocational College, Longdongbei Road 321, Tianhe District, Guangzhou 510520, China*

[6] *Graduate School of Science and Technology, Shizuoka University, 3-5-1 Johoku, Naka-ku, Hamamatsu 432-8561, Japan*

[*] Corresponding author. Ziyin Yang, Tel: +86-20-38072989, *Email address*: zyyang@scbg.ac.cn.

[+] These authors contributed equally to this work.

Supplementary Information

Table S1 Primers of qRT-PCR used in this study

Gene	Accession number	Forward primer 5'-3'	Reverse primer 5'-3'
LOX1	EU195885	GCTGACTGGACAACCGATGA	CAACATATGCTTCTATGAAAATGC
LOX2	FJ418174	GTTTTGTCAAATCATTCGGT	TTCTTCAAACTCAAGTTTG
LOX3	FJ794853	GGGACAACACTGTATGGG	CCAGAGTCATGAGCAAGGG
LOX4	HM370508	TTCTTGGCATTTCCCTCGTA	TCATCCTCGTTCATCCTTGT
PAL	D26596	ATTCCTTGCCAATCCTGTAA	ACTGCCTCGGCTGTCTTTCT
PAAS	FS952786	GGGAACCTTCAATCTTAC	ACATGCCTTTCTTCTGTC
PAR1	KR873393	TGGTTATTGGTCCTCTGTT	ATATCTTCCATTGGCTGAA
PAR2	KR873394	ACTGGTTATCGGTCCTCTC	GCATTGGCAACATCTTTA
TPS1	KF006849	TGAAGTTCGCTAGAGACCAACCTCTAA	TCCACAGCACCAAGTTCCCATCTA
TPS2	KR873395	ATTCTTAAAATGGACGGGCT	TGAGGACATCTTCGAACAAG
TPS3	KR873396	CAGCACAAACGAAATTTCCT	CATTCCATGACCCAAGAGAA
β-actin	HQ420251.1	GCCATATTTGATTGGAATGG	GGTGCCACAACCTTGATCTT

LOX, 9/13-lipoxygenase; *PAL, phenylalanine ammonialyase*; *PAAS, phenylacetaldehyde synthase*; *PAR, phenylacetaldehyde reductases*; *TPS, terpene synthase*.

Table S2 Primers used to clone the ORF of genes

Gene	Forward primer	Reverse primer
PAL	ACGCGTCGACAAATGGATAGTACC ACCGCC	TTAGCGGCCGCCTAACAGATAGGAAG AGG
TPS1	ACGCGTCGACAAATGCAAATCTTCCACT GTGC	TTAGCGGCCGCCTAAGGTAATTTATC ATAG
TPS3	ACGCGTCGACAAATGGCTGCCACC ACCACC	TTAGCGGCCGCTTAGAGAGAAACAGTATC AC

Table S3 Comparison of volatiles in 0 min and 120 min or 240 min treatments of dark, UVA, blue light, red light, and near-infrared (NIR) on postharvest tea leaves

	0 min	Dark	UVA	Blue	Red	NIR
			**120 min**			
Benzyl alcohol[a]	142.87 ±2.97ab	113.40 ±7.60c	135.80 ±6.96b	143.26 ±4.93ab	154.37 ±7.02a	156.82 ±2.92a
Terpineol, alpha-[b]	0.10 ±0.02a	0.04 ±0.00c	0.05 ±0.01bc	0.07 ±0.01bc	0.08 ±0.00b	0.06 ±0.00bc
Benzaldehyde[a]	10.69 ±2.05a	5.83 ±0.41b	7.27 ±0.97b	7.80 ±0.50b	7.57 ±0.99b	8.30 ±0.72b
3-Hexen-1-ol acetate [a]	20.50 ±1.08a	1.98 ±0.27c	2.06 ±1.08c	4.50 ±0.50bc	5.32 ±1.13b	3.02 ±1.67bc
Phenylethyl alcohol[a]	19.55 ±0.83ab	15.37 ±0.79c	19.27 ±1.67b	20.47 ±1.29ab	20.73 ±0.80ab	22.47 ±1.12a
Geraniol[a]	130.59 ±3.55a	104.46 ±6.05c	110.23 ±3.59bc	124.28 ±8.54ab	137.82 ±11.91a	121.55 ±6.33abc
Nerol[b]	0.05 ±0.00ab	0.04 ±0.01b	0.05 ±0.00ab	0.05 ±0.01ab	0.06 ±0.00a	0.06 ±0.00a
Nonanal[b]	0.08 ±0.01a	0.06 ±0.01c	0.06 ±0.01bc	0.08 ±0.01abc	0.08 ±0.00ab	0.07 ±0.01abc
1-Nonanol [b]	0.05 ±0.01bcd	0.04 ±0.00d	0.05 ±0.00cd	0.06 ±0.00ab	0.07 ±0.00a	0.07 ±0.01cd
3-Hexen-1-ol [a]	98.30 ±2.30bc	67.13 ±5.01e	79.37 ±6.15de	108.39 ±5.61ab	117.50 ±4.84a	89.70 ±9.62cd
Methyl salicylate[a]	52.71 ±6.48bc	45.26 ±3.70c	53.61 ±1.25bc	60.90 ±2.42ab	62.53 ±3.25a	55.79 ±0.45ab
Linalool[a]	76.16 ±2.75a	75.26 ±6.22a	75.23 ±2.06a	84.97 ±6.65a	82.78 ±2.58a	73.71 ±3.18a
Linalool oxide[a]	75.42 ±2.01a	75.06 ±7.40a	77.55 ±0.97a	85.95 ±7.09a	83.22 ±2.96a	75.90 ±5.95a
2,6-dimethyl-2,7-octad iene-1,6-diol [b]	0.11 ±0.01a	0.10 ±0.01a	0.12 ±0.01a	0.12 ±0.02a	0.10 ±0.01a	0.11 ±0.01a
Linalool pyran oxide[b]	0.41 ±0.02bc	0.39 ±0.02c	0.41 ±0.01abc	0.48 ±0.04ab	0.46 ±0.02a	0.45 ±0.02a
2-Hexenal [b]	0.50 ±0.03bc	0.40 ±0.03c	0.58 ±0.02b	0.79 ±0.10a	0.75 ±0.04a	0.45 ±0.03bc
Nerolidol 2[a]	6.28 ±0.20d	11.07 ±0.92c	12.94 ±0.61c	17.41 ±1.22b	21.59 ±1.17a	14.59 ±2.26c
2-Penten-1-ol [a]	14.34 ±2.65b	24.47 ±1.51a	21.68 ±4.51ab	25.33 ±3.17a	25.73 ±0.81a	25.70 ±1.18a
Benzeneacetaldehyde[b]	0.04 ±0.01b	0.04 ±0.01b	0.08 ±0.01a	0.08 ±0.01a	0.07 ±0.01a	0.07 ±0.01a

Indole[a]	0.00 ±0.00a	19.73 ±1.14a	27.37 ±0.68a	30.06 ±1.38a	28.88 ±1.22a	22.04 ±2.85a
			**240 min**			
Benzyl alcohol[a]	142.87 ±2.97a	136.83 ±4.62ab	126.20 ±7.78bc	119.64 ±2.96c	122.68 ±7.10c	137.28 ±8.92a
Terpineol, alpha-[b]	0.10 ±0.02a	0.06 ±0.01b	0.06 ±0.01b	0.05 ±0.01b	0.05 ±0.00b	0.06 ±0.00b
Benzaldehyde[a]	10.69 ±2.05a	8.24 ±0.87ab	8.22 ±0.79ab	6.99 ±0.73b	7.37 ±0.47b	8.72 ±1.37ab
3-Hexen-1-ol acetate [a]	20.50 ±1.08a	1.08 ±1.01c	2.16 ±1.03bc	2.83 ±0.23b	3.24 ±0.81b	0.84 ±1.19c
Phenylethyl alcohol[a]	19.55 ±0.83ab	19.86 ±1.46a	17.90 ±0.71bc	16.41 ±0.56c	14.39 ±0.87d	20.33 ±0.62a
Geraniol[a]	130.59 ±3.55ab	138.47 ±18.77a	121.68 ±3.73abc	108.99 ±3.87bc	100.37 ±0.98c	139.67 ±4.75a
Nerol[b]	0.05 ±0.00ab	0.05 ±0.01a	0.04 ±0.00ab	0.05 ±0.00ab	0.04 ±0.00b	0.05 ±0.00ab
Nonanal[b]	0.08 ±0.01ab	0.10 ±0.01ab	0.08 ±0.01b	0.07 ±0.00b	0.07 ±0.01b	0.11 ±0.03a
1-Nonanol [b]	0.05 ±0.01ab	0.06 ±0.00ab	0.06 ±0.01ab	0.05 ±0.00b	0.05 ±0.00b	0.06 ±0.01a
3-Hexen-1-ol [a]	98.30 ±2.30b	89.50 ±4.09b	97.17 ±4.70b	100.66 ±7.40ab	99.94 ±6.00b	110.14 ±4.66a
Methyl salicylate[a]	52.71 ±6.48ab	45.42 ±0.43b	50.96 ±1.73ab	51.59 ±2.62ab	53.18 ±1.27ab	59.02 ±2.49a
Linalool[a]	76.16 ±2.75c	87.30 ±2.25b	106.48 ±3.28a	108.76 ±5.76a	119.76 ±3.52a	112.53 ±4.93a
Linalool oxide[a]	75.42 ±2.01b	70.35 ±2.55b	85.84 ±5.82a	86.12 ±3.90a	91.34 ±5.53a	91.43 ±2.70a
2,6-dimethyl-2,7-octad iene-1,6-diol [b]	0.11 ±0.01a	0.11 ±0.03a	0.15 ±0.01a	0.14 ±0.01a	0.16 ±0.00a	0.16 ±0.05a
Linalool pyran oxide[b]	0.41 ±0.02b	0.44 ±0.03ab	0.46 ±0.02ab	0.45 ±0.01ab	0.44 ±0.01b	0.51 ±0.03a
2-Hexenal [b]	0.50 ±0.03c	0.59 ±0.07b	0.56 ±0.03bc	0.57 ±0.04bc	0.54 ±0.02bc	0.70 ±0.07a
Nerolidol 2[a]	6.28 ±0.20c	18.43 ±0.93ab	18.06 ±0.83ab	15.64 ±0.33b	17.47 ±2.38ab	22.49 ±3.07a
2-Penten-1-ol [a]	14.34 ±2.65d	28.59 ±1.74c	33.02 ±1.68bc	33.52 ±2.48abc	36.97 ±0.11ab	38.86 ±4.69a
Benzeneacetaldehyde[b]	0.04 ±0.01c	0.10 ±0.01a	0.09 ±0.01ab	0.06 ±0.01bc	0.11 ±0.03ab	0.10 ±0.02a
Indole[a]	0.00 ±0.00a	107.09 ±14.77a	108.57 ±2.41a	92.02 ±5.16a	96.32 ±3.14a	96.34 ±2.38a

[a], nmol/g; [b], peak area ratio of analyte to internal standard per g. The data are expressed as mean ± S.D. Different means with different letters in the same row are significantly different from each other ($p \leq 0.05$).

Table S4 Comparison of metabolites (nmol/g) in 0 min and 240 min treatments of dark, UVA, blue light, red light, and near-infrared (NIR) on postharvest tea leaves

	0 min	Dark-240 min	UVA-240 min	Blue-240 min	Red-240 min	Near-infrared (NIR)-240 min
Gly	99.90 ±5.78a	88.20 ±1.11b	100.26 ±4.95a	96.83 ±2.91ab	96.82 ±2.67ab	96.30 ±3.01ab
Ornithine	3.42 ±0.11ab	2.32 ±0.16c	4.18 ±0.42a	2.85 ±0.11bc	3.40 ±0.56ab	2.26 ±0.22c
γ-Aminobutyric acid	438.25 ±24.24b	342.32 ±18.82c	453.08 ±31.97b	580.99 ±28.94a	450.00 ±20.86b	371.38 ±29.42c
Ser	2535.56 ±87.01ab	2365.43 ±41.72b	2534.34 ±95.19ab	2720.75 ±113.64a	2551.60 ±23.51ab	2408.53 ±42.51b
Ala	1039.04 ±43.83ab	962.29 ±49.52b	974.35 ±25.80b	1098.84 ±29.69a	975.35 ±18.33b	964.72 ±36.30b
Citrulline	47.76 ±1.80b	47.83 ±5.30b	46.20 ±0.73b	64.75 ±2.43a	44.47 ±2.70b	46.22 ±5.15b
Asp	3401.65 ±140.54c	4676.28 ±117.44a	4350.30 ±214.52ab	4222.21 ±145.44b	3742.34 ±99.33c	4529.79 ±81.95ab
Anthranilic acid	0.27 ±0.33b	1.17 ±0.17a	0.30 ±0.37b	0.66 ±0.41ab	0.27 ±0.33b	0.91 ±0.03ab
Tyramine	1.80 ±1.15a	1.84 ±0.20a	2.00 ±0.22a	1.81 ±0.19a	1.37 ±0.19a	2.07 ±0.20a
Gln	12759.28 ±626.89a	13502.28 ±1425.89a	11259.01 ±1126.09a	10784.94 ±1043.29a	10978.79 ±907.32a	12125.54 ±1273.26a
Arg	4261.36 ±36.54bc	4948.64 ±374.40b	4953.73 ±254.62b	5773.23 ±200.76a	3934.72 ±147.23c	4570.85 ±432.65bc
Hydroxyproline	62.00 ±0.98c	69.68 ±2.62c	81.26 ±2.02ab	94.37 ±14.37a	83.43 ±0.50ab	86.57 ±8.60ab
Glu	5446.66 ±126.60b	5993.67 ±137.81ab	5927.44 ±313.79ab	6184.45 ±209.26a	5934.57 ±167.05ab	5991.33 ±193.90ab
Met	3.56 ±0.52b	6.55 ±0.16a	5.92 ±0.36a	6.82 ±0.74a	5.95 ±0.26a	6.36 ±0.76a
His	126.17 ±6.61c	195.65 ±17.11ab	192.21 ±3.02ab	215.59 ±8.40a	172.19 ±9.07b	180.92 ±17.69b
Thr	540.97 ±5.44b	722.27 ±28.74a	746.69 ±23.07a	753.34 ±5.65a	735.04 ±38.56a	703.74 ±42.60a
S-Adenosylmethionine	16.10 ±0.71c	26.63 ±1.47b	29.72 ±0.83a	29.75 ±0.32a	26.61 ±1.19b	25.67 ±0.62b
Asn	221.83 ±12.46c	376.11 ±27.57b	471.33 ±18.65a	477.93 ±7.59a	392.22 ±19.10b	406.69 ±58.26ab
Trp	162.91 ±6.62c	375.32 ±13.92b	409.94 ±17.23ab	435.61 ±14.08a	431.82 ±10.13a	404.00 ±27.50ab

β-Ala	44.09 ±1.20c	146.72 ±3.67a	146.74 ±10.54a	145.18 ±1.16ab	128.49 ±3.88b	142.44 ±10.82ab
Lys	96.02 ±0.67c	270.04 ±10.14b	331.12 ±19.14a	336.09 ±13.84a	276.33 ±7.34b	266.04 ±19.39b
Phe	42.03 ±1.57c	162.61 ±2.52b	206.90 ±9.96a	216.80 ±22.70a	205.88 ±5.50a	169.69 ±11.71b
Pro	115.21 ±2.68c	253.02 ±10.09b	329.40 ±39.25a	357.78 ±24.52a	300.08 ±3.95ab	303.36 ±44.51ab
Val	87.96 ±2.92c	280.92 ±9.98b	376.95 ±36.29a	386.33 ±18.50a	338.78 ±8.43ab	324.68 ±38.09ab
Ile	36.24 ±0.89c	165.32 ±8.30b	222.64 ±29.29a	231.97 ±13.65a	205.06 ±11.94ab	197.05 ±26.25ab
Leu	35.82 ±1.07c	263.99 ±15.90b	351.87 ±36.43a	361.21 ±23.03a	306.09 ±16.80ab	303.22 ±39.53ab
Tyr	38.51 ±1.12d	382.41 ±26.63c	496.55 ±37.44ab	581.46 ±33.29a	482.65 ±32.79b	437.13 ±52.42bc
Uridine	10.40 ±1.26ab	13.81 ±0.87a	10.89 ±0.91ab	11.52 ±1.77ab	12.17 ±0.90ab	9.87 ±1.05b
Cytosine	1.00 ±0.00	1.13 ±0.16	1.26 ±0.00	1.21 ±0.00	1.15 ±0.00	0.89 ±0.07
Inosine	2.62 ±0.37a	2.12 ±0.22ab	1.78 ±0.15ab	1.94 ±0.25ab	1.51 ±0.55b	1.40 ±0.34b
Glutathione (GSH)	3.93 ±0.13a	3.50 ±0.17a	3.50 ±0.18a	3.38 ±0.19a	2.60 ±0.28b	3.39 ±0.32a
Adenosine	5.68 ±0.45c	5.56 ±0.12c	7.50 ±0.75b	7.61 ±0.26b	9.23 ±0.31a	6.36 ±0.22c
Guanosine	11.48 ±1.08c	17.75 ±0.15b	19.09 ±0.96ab	18.98 ±0.96ab	20.35 ±0.31a	18.99 ±0.66ab
Glutathione (GSSG)_divalent	84.67 ±3.55b	124.80 ±15.57a	138.57 ±14.07a	141.74 ±1.82a	137.43 ±16.74a	133.05 ±16.68a
Cytidine	4.04 ±0.08c	11.93 ±0.32b	12.17 ±0.30b	11.77 ±0.44b	13.69 ±0.37a	11.39 ±0.57b
3-Phosphoglyceric acid	90.01 ±1.72a	81.05 ±3.10b	81.87 ±3.85b	92.55 ±3.81a	80.67 ±2.17b	82.54 ±0.16b
Malic acid	3206.21 ±120.50a	2195.19 ±89.19bc	2115.48 ±42.99c	2443.27 ±101.75c	2791.38 ±120.72b	2289.80 ±110.84bc
Fumaric acid	138.97 ±8.80a	136.02 ±7.23a	129.02 ±1.59a	134.46 ±2.99a	120.98 ±11.38a	137.38 ±10.55a
2-Oxoisovaleric acid	1.46 ±0.20a	0.29 ±0.35bc	0.98 ±0.60ab	0.00 ±0.00c	0.29 ±0.36bc	0.00 ±0.00c
Citric acid	2360.98 ±74.40a	2452.78 ±62.55a	2265.59 ±68.59a	2345.01 ±56.02a	2346.39 ±182.21a	2221.47 ±88.44a
Isocitric acid	133.89 ±7.57ab	139.58 ±3.58a	133.92 ±6.64ab	140.55 ±8.43a	142.03 ±7.30a	119.04 ±5.35b

Salicylic acid	66.71 ±4.58abc	65.28 ±2.18bc	61.54 ±1.10c	68.56 ±1.93ab	72.86 ±1.91a	71.29 ±0.37ab
Phenylpyruvic acid	3.51 ±0.48a	3.47 ±0.10a	3.29 ±0.16a	3.36 ±0.12a	3.92 ±0.08a	3.87 ±0.49a
Glycolic acid	37.94 ±46.47b	0.00 ±0.00b	56.11 ±68.73b	240.05 ±18.79a	0.00 ±0.00b	0.00 ±0.00b
Lactic acid	170.39 ±10.59a	96.91 ±10.54a	213.61 ±88.37a	127.54 ±10.62a	342.86 ±241.21a	112.70 ±6.32a
Phosphoenolpyruvic acid	8.04 ±0.53b	8.27 ±0.08b	9.62 ±2.39ab	12.71 ±1.16a	11.88 ±0.60a	11.11 ±0.62ab
cis-Aconitic acid	49.40 ±4.22c	47.93 ±0.59c	52.77 ±2.38c	68.26 ±3.70a	64.83 ±6.36ab	56.87 ±2.23bc
Benzoic acid	3.27 ±0.32b	3.54 ±0.07b	3.48 ±0.20b	3.69 ±0.07ab	4.09 ±0.23a	3.53 ±0.11b
Quinic acid	15317.35 ±868.21a	15998.29 ±1534.98a	15624.38 ±1454.36a	16164.08 ±1781.93a	18436.31 ±652.13a	15815.53 ±2605.23a
Succinic acid	688.88 ±24.81b	714.88 ±8.16ab	698.89 ±35.34b	748.31 ±26.92ab	795.80 ±55.88a	684.12 ±18.83b
Gluconic acid	317.51 ±12.21c	359.69 ±16.38b	355.53 ±13.01b	409.21 ±5.02a	406.16 ±18.93a	361.79 ±17.85b
Shikimic acid	461.14 ±18.90c	533.91 ±9.54ab	517.98 ±2.11ab	497.18 ±12.01bc	536.13 ±21.69ab	546.10 ±26.77a
Chorismic acid	6.59 ±4.07a	10.23 ±0.50a	10.78 ±0.65a	7.40 ±4.57a	8.25 ±5.06a	12.51 ±0.42a
3-Phosphoglyceric acid	90.01 ±1.72a	81.05 ±3.10b	81.87 ±3.85b	92.55 ±3.81a	80.67 ±2.17b	82.54 ±0.16b
Fructose 6-phosphate	68.96 ±1.42a	54.52 ±2.87bc	47.95 ±1.89c	52.13 ±1.94c	61.64 ±6.10ab	54.95 ±2.52bc
Fructose 1,6-diphosphate	21.85 ±0.68a	15.55 ±0.82b	15.26 ±1.32b	16.58 ±0.53b	16.72 ±0.14b	15.59 ±0.55b
6-Phosphogluconic acid	9.95 ±0.62a	5.66 ±0.42c	7.72 ±1.00b	8.08 ±0.20b	8.35 ±0.42ab	7.82 ±0.21b
Pyruvic acid	26.02 ±3.32a	18.23 ±0.31b	20.60 ±0.97b	17.68 ±0.10b	21.58 ±2.81ab	21.75 ±0.84ab
Glucose 6-phosphate	402.83 ±13.00b	418.33 ±5.27ab	390.19 ±8.16b	419.30 ±34.11ab	451.33 ±14.20a	378.83 ±10.38b
Glucose 1-phosphate	27.64 ±1.47ab	25.48 ±1.39b	25.39 ±0.14b	25.91 ±0.95b	30.18 ±1.96a	24.30 ±0.62b
Dihydroxyacetone phosphate	12.28 ±0.09ab	9.84 ±0.35c	10.57 ±1.02bc	13.84 ±1.40a	13.69 ±0.58a	12.02 ±0.91abc
Ribose 5-phosphate	2.40 ±0.17b	1.84 ±0.15c	2.59 ±0.20ab	2.82 ±0.11ab	2.97 ±0.10a	2.60 ±0.31ab
Ribulose 5-phosphate	11.48 ±1.25ab	9.71 ±0.41b	11.26 ±1.44ab	13.67 ±0.63a	13.09 ±1.54a	12.75 ±0.83a

Glycerol-3-phosphate	64.08 ±5.24b	80.59 ±4.23ab	88.16 ±11.12a	95.82 ±6.60a	88.97 ±1.76a	87.44 ±11.56a
Sedoheptulose 7-phosphate	22.28 ±1.10d	28.59 ±3.17c	39.38 ±3.00ab	37.88 ±2.67ab	42.03 ±1.16a	33.92 ±2.29bc
Gluconic acid	317.51 ±12.21c	359.69 ±16.38b	355.53 ±13.01b	409.21 ±5.02a	406.16 ±18.93a	361.79 ±17.85b
Phosphoenolpyruvic acid	8.04 ±0.53b	8.27 ±0.08b	9.62 ±2.39ab	12.71 ±1.16a	11.88 ±0.60a	11.11 ±0.62ab

The data are expressed as mean ± S.D. Different means with different letters in the same row are significantly different from each other ($p \leq 0.05$).

Table S5 Full name of the metabolites in tea leaves.

Abbreviation	Full name
Gly	Glycine
Ser	Serine
Ala	Alanine
Asp	Aspartic acid
Arg	Arginine
Glu	Glutamic acid
His	Histidine
Thr	Threonine
Asn	Asparagine
Trp	Tryptophan
β-Ala	β-Tryptophan
Lys	Lysine
Phe	Phenylalanine
Pro	Proline
Val	Valine
Ile	Isoleucine
Leu	leucine
Tyr	Tyrosine

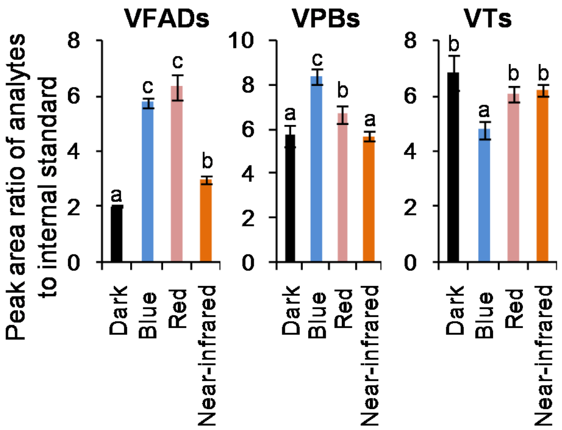

Figure S1 Effects of LED and dark on volatiles of preharvest tea leaves after treatment from the 1st leaf stage to the 4th leaf stage

VFADs, volatile fatty acid derivatives; VPBs, volatile phenylpropanoids/ benzenoids; VTs, volatile terpenes. Different means with different letters are significantly different from each other ($p \leq 0.05$).

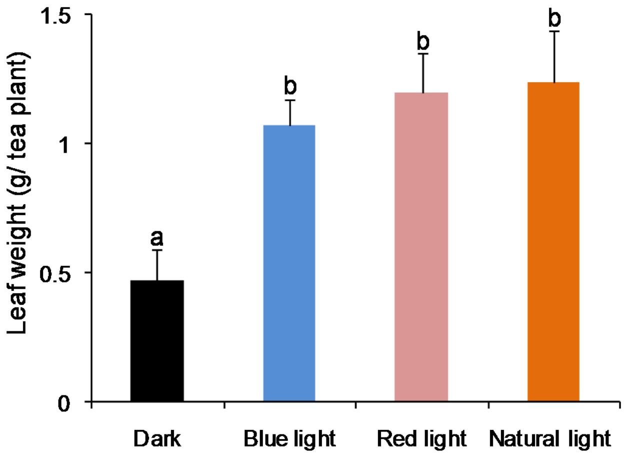

Figure S2 Effects of LED, dark, and natural light on biomass of preharvest tea leaves after treatment from the 1st leaf stage to the 4th leaf stage

Tea seedlings at the 1st leaf stage were treated with blue light, red light, and dark, respectively. After 14 days-treatments, the tea plants developed from the 1st leaf stage to 4th leaf stage, and the weights of growing four leaves from each treatment were analyzed. In contrast to natural light treatment as a control, blue and red lights treatments had no significant effects on the biomass of tea leaves, whereas dark treatment significantly reduced the biomass of tea leaves. Different means with different letters are significantly different from each other ($p \leq 0.05$).

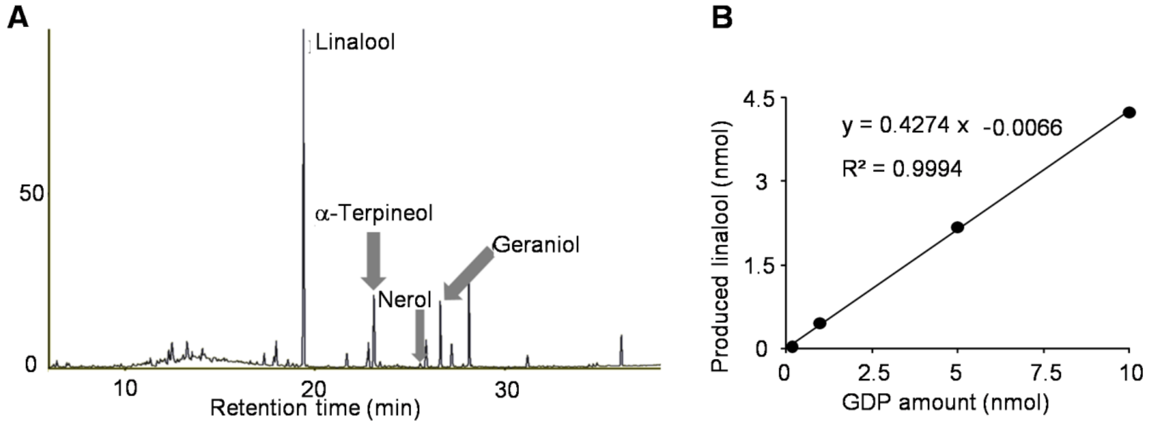

Figure S3 GC-MS chromatogram of products from acidic hydrolysis of GDP (A) and linear relationships between GDP amount and its acidic hydrolyzed product linalool content (B). GDP, geranyl diphosphate. (A) Fifty nmol of GDP was used for acidic hydrolysis reaction.

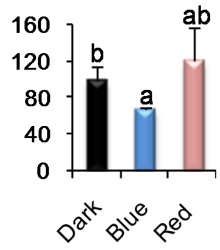

Figure S4 Effects of blue light and red light on the content of linoleic acid in preharvest tea leaves. All treatments on preharvest tea leaves were carried out for 3 days. The y-axis unit is relative content (%) of metabolites, which was calculated based on the dark treatment (100%) as a control. Different means with different letters are significantly different from each other ($p \leq 0.05$).

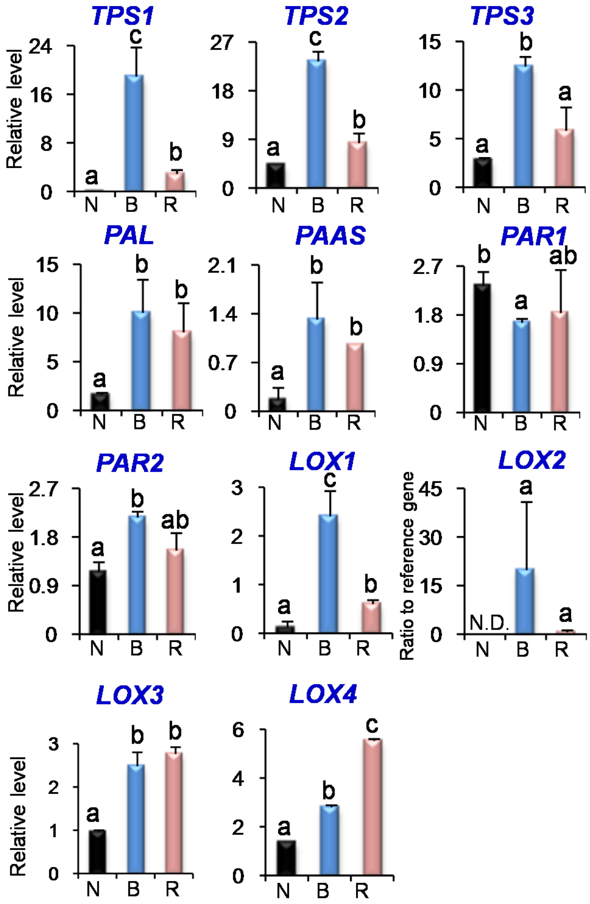

Figure S5 Effects of blue light and red light on the expression levels of key genes involved in formation of volatiles in preharvest tea leaves

N, natural light treatment. B, blue light treatment. R, red light treatment. N.D., not detected. *TPS, terpene synthase*. *PAL, phenylalanine ammonialyase*. *PAAS, phenylacetaldehyde synthase*. *PAR, phenylacetaldehyde reductases*. *LOX, 9/13-lipoxygenase*. All treatments on preharvest tea leaves were carried out for 3 days. The relative expression levels of genes were calculated based on the dark treatment (1) as a control. Different means with different letters are significantly different from each other ($p \leq 0.05$).

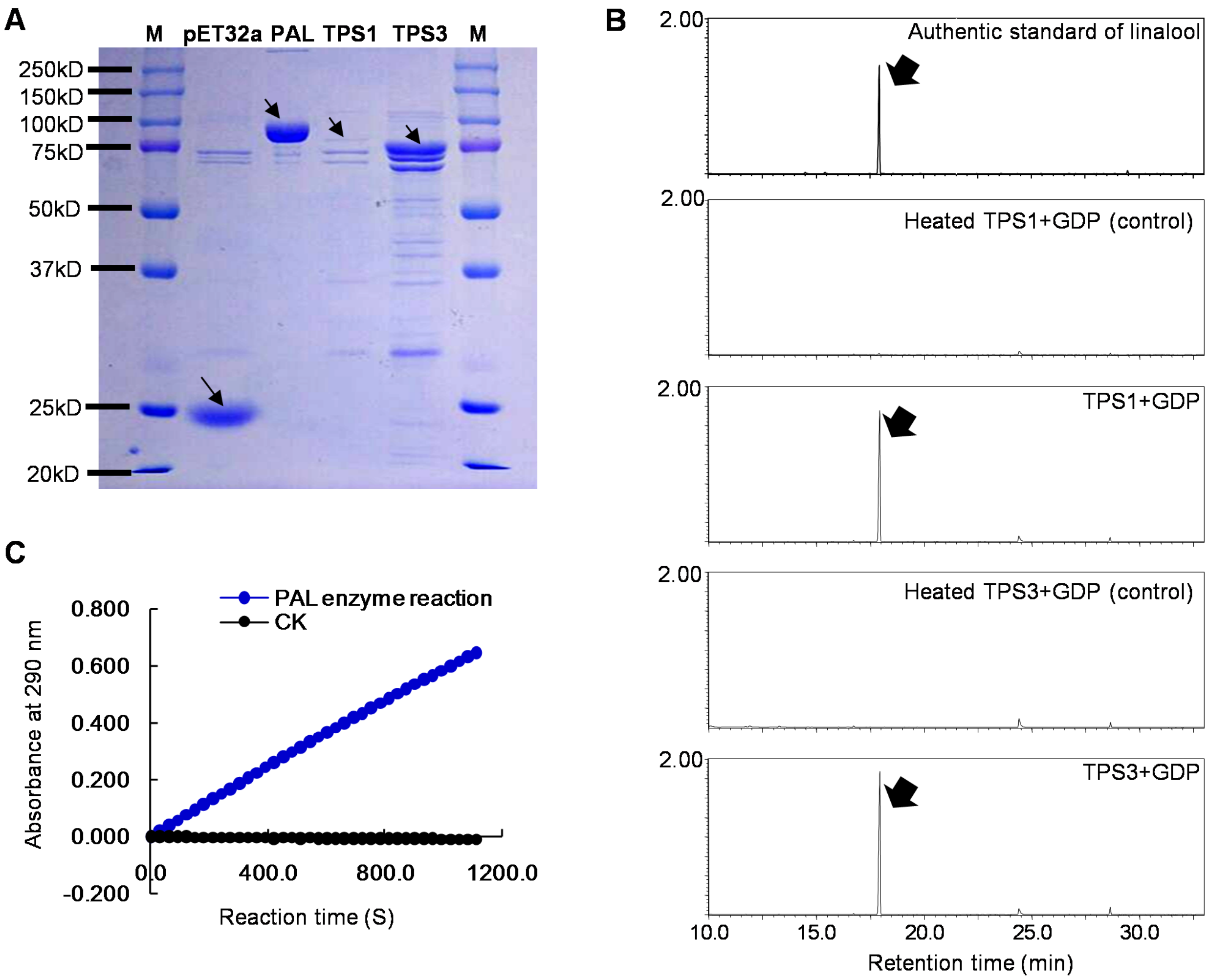

Figure S6 SDS-page identification (A) and functional identifications (B and C) of TPS1, TPS3, and PAL recombinant proteins produced in *Escherichia coli.*

TPS, terpene synthase; PAL, phenylalanine ammonialyase; GDP, geranyl diphosphate. (A) Denatured 6×His-PAL, TPS1, and TPS3 recombinant proteins were separated on a 10% acrylamide SDS-PAGE gel. The marker lane shows the denatured protein molecular weight markers with the size label on the left. The proteins were indicated by the arrows. (B) The GC-MS chromatograms indicate that *TPS1* and *TPS3* recombinant protein produced in *E.coli* exhibited the activity of transformation from GDP to linalool. (C) The reaction product *trans*-cinnamic acid (CA) was determined at 290 nm. The PAL was assayed from the increase in absorbance at 290 nm, suggesting that the PAL recombinant protein produced in *E. coli* exhibited the activity of transformation from L-Phe to CA.

```
PhPAAS          CAGATCATTGAAGTTGTGGCTTGTGTTAAAAAGTTATGGTGTAGCTAACCTCAGAAATTT
CsPAAS          -----------------------------------------GGGGGAACCTTCAATCTTA
RhPAAS          TCGGGCATTGAAGCTGTGGCTTGTGCTAAGAAGCTATGGTGTGGCTAACCTTAGAAACTT
                                                           *  *****   *   *

PhPAAS          CATAAGAAGCCATATAGAAATGGCTAAGCATTTTGAAGAACTTGTAGCCATGGATGAGAG
CsPAAS          CATAAGAAACCATATTGAATTGGCTAAACACTTTGAAGAGCTTGTTTCTCAAGACCCGAG
RhPAAS          TATTCGTATCCATGTCAAAATGGCCAAGACTTTTGAAGGGCTTGTGAGAATGGACAAGAG
                 **  * * **** *  ** **** **    *******  *****       **   ***

PhPAAS          GTTCGAAATTATGGCCCCAAGAAATTTCTCTCTGGTGTGCTTTAGGGTTTCACTATTGGC
CsPAAS          ATTTCAGGTTGTTGCTCCTCGGAAGTTTTCATTGGTTTGTTTTTGCCTTCT---------
RhPAAS          GTTTGAGATTCTGGTGCCTAGAAACTTCTCCTTGGTCTGCTTTAGAATTTCACCATCGGC
                 **  *  ** * *  **  * ** ** **  **** ** *** *  **

PhPAAS          ATTGGAGAAAAAGTTTAACTTTGTCGATGAAACCCAAGTGAATGAGTTCAACGCTAAGCT
CsPAAS          ------------GCCTCCCCATAACAACGAAGACTGTGCTAACAAACTAAACCATGACCT
RhPAAS          CTTGATCAGTAGTAATGAGGATGATGAGATCGGTATGGTAAACGAGGTCAATTGCAAATT
                               *     *    *          *  **  *  * **     *  *

PhPAAS          TTTAGAGTCCATTATTTCATCCGGTAATGTTTACATGACTCATACTGTGGTTGAAGGAGT
CsPAAS          ACTGGATGCTGTTAACTCAACCGGGAAATTATTCATTTCTCATACAGTTCTATCAGGTAA
RhPAAS          GCTGGAGGCCATCAATGCATCAGGTAAAGCATACATGACTCATGCTGTGGTTGGAGGGCT
                  * **  *  * *   ** * ** **    * ***  ***** * **  *   ***

PhPAAS          TTACATGATACGTTTTGCTGTTGGTGCACCTCTCACTGACTATCCCCATATTGATATGGC
CsPAAS          GTACACATTACGCTTTGCAGTAGGAGCTCCATTGACAGAAGAAAGGCATGTAAATGCAGA
RhPAAS          GTACGTGCTTCGTTGCGCGGTTGGTGCAACTCTGACCGAGGAAAAGCACATAGTCGAGGC
                 ***    * ** *  ** ** ** **  *  * ** **  *    **  *      *
```

Figure S7 Alignment of *Camellia sinensis PAAS* (*CsPAAS*) with *Petunia x hybrida PAAS* (*PhPAAS*, DQ243784) and rose hybrid *PAAS* (*RhPAAS*, DQ192639).

cDNA sequences were aligned using Clustal X. Method: tea plant shoot stems cDNA library from NCBI was chosen to blast, one PAAS (phenylacetaldehyde synthase) unigenes (*CsPAAS*, FS952786) was obtained by blasting with the homologous sequences from *PhPAAS* and *RhPAAS*. *Petunia x hybrida PAAS* and rose hybrid *PAAS* from paper: *Kaminaga, Y., et al., Plant phenylacetaldehyde synthase is a bifunctional homotetrameric enzyme that catalyzes phenylalanine decarboxylation and oxidation. Journal of Biological Chemistry, 2006, 281, 23357-23366.*

```
CsPAR1      AAAAGAGTGGTTTTGACATCGTCTGTAGCTGCAGTTGCATACAATGGTAGGCCTCGAACT
CsPAR2      AAAAGAGTAGTGTTGACATCTTCTGTAGCTGCCGTTGCTTTCAATGGTAGGCCTCGAGCT
RdPAR       AAACGGGTGGTCTTAACATCTTCTATAGCCGCAGTTGCATATAATGGAAAGCCTCGAACT
SlPAR1      AAACGAGTTGTTTTAACGTCTTCCATAGCTGCAGTTGCTTACAGTGGTCAGCCTCGGACA
SlPAR2      AGAAGAGTGGTCTTGACATCATCTGTTGCAGCAGTTGCTTTCAATGGCAAGCCAAGAACC
            * * * ** ** ** ** ** **  * ** ** ***** *  * ***   ***  *  *

CsPAR1      TCTGATGTGATAATTGATGAGACTTGGTTTTCTGATCCGGTGTCATGCAAGGAAAATAAG
CsPAR2      CCTGACGTCGTAGTTGATGAGAGTTGGTTTTCTGATCCAGAGTTCTGCAAGCAAAATAAG
RdPAR       CCTGATGTAGTGGTTGATGAGACTTGGTTTACTGATCCAGATGTCTGTAAGGAATCGAAG
SlPAR1      CCTGAGGTTGTGGTTGATGAGAGCTGGTGGACCAGTCCAGACTACTGCAAAGAAAAACAG
SlPAR2      CCTGAAGTGGTGGTTGATGAAACATGGTGGTCAGATCCTGACTTTTGCAGAGAATCACAG
             **** **  *  ******* *  ****   *   *** *     ** *   **    **

CsPAR1      CTTTGGTATCAGCTGTCCAAGACTCTAGCAGAGGATGCTGCCTGGAAGTTTGCAAAAGAG
CsPAR2      CTTTGGTATGTGCTGTCAAAGACTTTAGCAGAGGATGCTGCCTGGAAGTTTACAAAAGGA
RdPAR       CTATGGTATGTGCTTTCAAAGACTTTGGCGGAGGATGCTGCCTGGAAATTTGTAAAGGAG
SlPAR1      CTCTGGTATGTCCTCTCAAAGACATTGGCTGAGGATGCTGCGTGGAAGTTTGTGAAGGAG
SlPAR2      CTCTGGTATGTGCTTTCGAAGACATTAGCTGAGGATGCTGCGTGGAAGTTTGTGAAAGAG
            ** ******   ** ** *****  * ** *********** ***** ***   ** *

CsPAR1      AACGGTATTGACATGGTTGCAATAAACCCAGCAATGGTTATTGGTCCTCTGTTACAACCA
CsPAR2      AAGGGTATTGACATGGTTACAATTAACCCAGCAATGGTTGTCGGTCCTCTCTTGCAGCCA
RdPAR       AAGGGAATTGACATGGTTACAATTAATCCTGCAATGGTGATCGGTCCTCTGTTACAGCCA
SlPAR1      AAAGGCATTGATATGGTTGTAGTAAACCCTGCTATGGTTATTGGTCCTCTGTTACAGCCT
SlPAR2      AAAGCTTTCGATATGGTTACAATAAACCCAGCAATGGTTATAGGCGGTTTGTTGCAACCA
            ** *   * ** ******  * * ** ** ** *****  * **   * * ** ** **

CsPAR1      ACACTTAATACAAGTTCTGCTGCTATCTTGAACTTAATAAATGGTTCACAAACATATCCA
CsPAR2      ACTCTCAATACAAGTGCTGCTGCAATTTTGAACGTAATAAATGGTTCACAAACATTTCCA
RdPAR       ACGCTAAATACAAGTGCTGCTGCAATTCTGAATATTATTAAGGGAGCTCGAACATATCCA
SlPAR1      ACACTTAATACCAGTTCTGCTGCAGTCTTGAGCTTGGTAAATGGTGCTGAGACATACCCA
SlPAR2      ACGCTTAATACAAGTGCTGCTGCTATCTTACAACTGCTAAATGGTTCTGAAACATACCCA
            ** ** ***** *** *******  *  *     *  * ** **  *    ****  ***

CsPAR1      AATGCTTCATTTGGATGGATTAATGTTAAAGATGTTGCAAATGCGCACATTCAAGCATAT
CsPAR2      AATTCTACATTTGGATGGGTTAATGTTAAAGATGTTGCCAATGCACATATTCAAGCATTT
RdPAR       AATGCAAGTTTTGGATGGATTAATGTCAAAGATGTTGCCAACGCACATGTTCAAGCATTT
SlPAR1      AATTCCTCTTTTGGGTGGGTTAACGTGAAAGATGTTGCAAATGCACATATTCTTGCATTT
SlPAR2      AATTCTACATTTGGTTGGGTTAACGTGAAAGATGTCGCCCTTGCACATATTCTGGCATTT
            *** *    ***** *** **** ** ******** **    ** **  ***  **** *

CsPAR1      GAGATTCCTTCAGCCAATGGAAGATATTGTTTGGTTGAGAGAGTCGCACACTACTCTGAA
CsPAR2      GAGATTCCTTCAGCTAATGGAAGATATTGTTTGGTTGAGAGCGTTGCACACTACTCTGAA
RdPAR       GAGATTCCTTCAGCTAGTGGAAGATATTGTTTAGTCGAGAGAGTTGCACACTTCACTGAA
SlPAR1      GAGAACCCTTCAGCTAATGGGAGATACTTAATGGTTGAGAGGGTTGCACACTATTCTGAT
SlPAR2      GAAAACCCTTCAGCTAATGGTAGATATTTAATGGTGGAGTCAGTTGCACACTACTCTGAG
```

```
                ** *  ******** * *** ***** *   * ** ***   ** *******   ****

CsPAR1          GTTGTGAACATACTACACAAGCTTTATCCTTCTTTTCAACTTCCAGAAAAGTCTGCGGAT
CsPAR2          GTTGTGAAGATACTACAAGAGCTGTTTCCTGCTTTTCAACTTCCAGAAAAGTGTGCTGAT
RdPAR           GTTCTGCAAATTATACATGAGCTGTACCCTGATTTGCAACTTCCAGAGAAATGTTCGGAT
SlPAR1          ATATTGAAGATATTGCGTGACCTTTATCCTACTATGCAACTTCCAGAAAAGTGTGCTGAT
SlPAR2          ATAGTTAAGATATTACGCGAGCTTTACCCTACACTGAAGCTTCCAGAAAAGTGTGCTGAT
                 *  *  * **  * *   * ** *  ***    *  * ******** ** * * * ***

CsPAR1          GACAAACCATTTATGCCAACATACCAGGTATCCAGGGAAAAAGCAAAAACTTTAGGAATT
CsPAR2          GACAAGCCATTTACGCCGACTTACCAGGTGTCTAAGGAAAGAACAAAAAGCTTAGGTATT
RdPAR           GATAAACCTTTTGTGCCAACATATCAGGTGTCCAAAGAAAAGGCAAAGAGCTTGGGAATT
SlPAR1          GACAACCCATTGATGCAAAATTATCAAGTATCAAAGGAGAAGGCAAAAAGCTTGGGTATT
SlPAR2          GATAAGCCATTTACGCCAACGTACCAGGTTAACGTAGAAAGAGCCAAAAAATTGGGTATT
                ** ** ** **   **  *  ** ** **       ** *   * ** *  ** ** ***

CsPAR1          GACTTCATTTCCCTCGAGGAGGGCCTCAAGGAAACTGTTGAAAGCTTGACAGAGAAGAAA
CsPAR2          GAGTTCATTCCCCTCAAGCAGAGCATCAAGGAAACAGTTGAAAGCTTGATGGAGAAGAAA
RdPAR           GAGTTTATTCCATTAGACATTAGCCTCAAGGAAACAATTGAAAGCTTGAAGGAAAAGAGT
SlPAR1          GAGTTTACTACCCTTGAAGAAAGCATCAAAGAAACTGTTGAAAGTTTGAAGGAAAAGAAG
SlPAR2          GAATTCATTCCTTTGGCGGAAAGCGTCAAGGAAACAGCTGAAAGCTTGAAAGAGAAGAAG
                ** ** * * *  *        ** **** *****   ****** ****  ** ****

CsPAR1          TTTTTTGGTATGTG-------------
CsPAR2          CTTTTCACTCGGTGA------------
RdPAR           ATCGTCAGCTTCTGA------------
SlPAR1          TTTTTTGGAGGTTCATCTTCTATGTAA
SlPAR2          TTTTACTGA------------------
                 *
```

Figure S8 Alignment of *Camellia sinensis PARs* (*CsPAR1* and *CsPAR2*) with tomato *PARs* (*SlPAR1*, NM_001247894; *SlPAR2*, NM_001247901) and *Rosa damascene PAR* (*RdPAR*, AB426519).

cDNA sequences were aligned using Clustal X. Method: Tea library of GAAC01.1 was downloaded from NCBI, two *PAR* (*Phenylacetaldehyde reductase*) unigenes (*CsPAR1* and *CsPAR2*) were obtained by blasting with the homologous sequences from *SlPAR1*, *SlPAR2*, and *RdPAR*.

Rosa damascene PAR from paper: *Chen, X.M., et al., Functional characterization of rose phenylacetaldehyde reductase (PAR), an enzyme involved in the biosynthesis of the scent compound 2-phenylethanol. Journal of Plant Physiology, 2011, 168, 88-95.*

Tomato *PARs* from paper: *Tieman, D.M., et al., Tomato phenylacetaldehyde reductases catalyze the last step in the synthesis of the aroma volatile 2-phenylethanol. Phytochemistry, 2007, 68,2660-2669.*

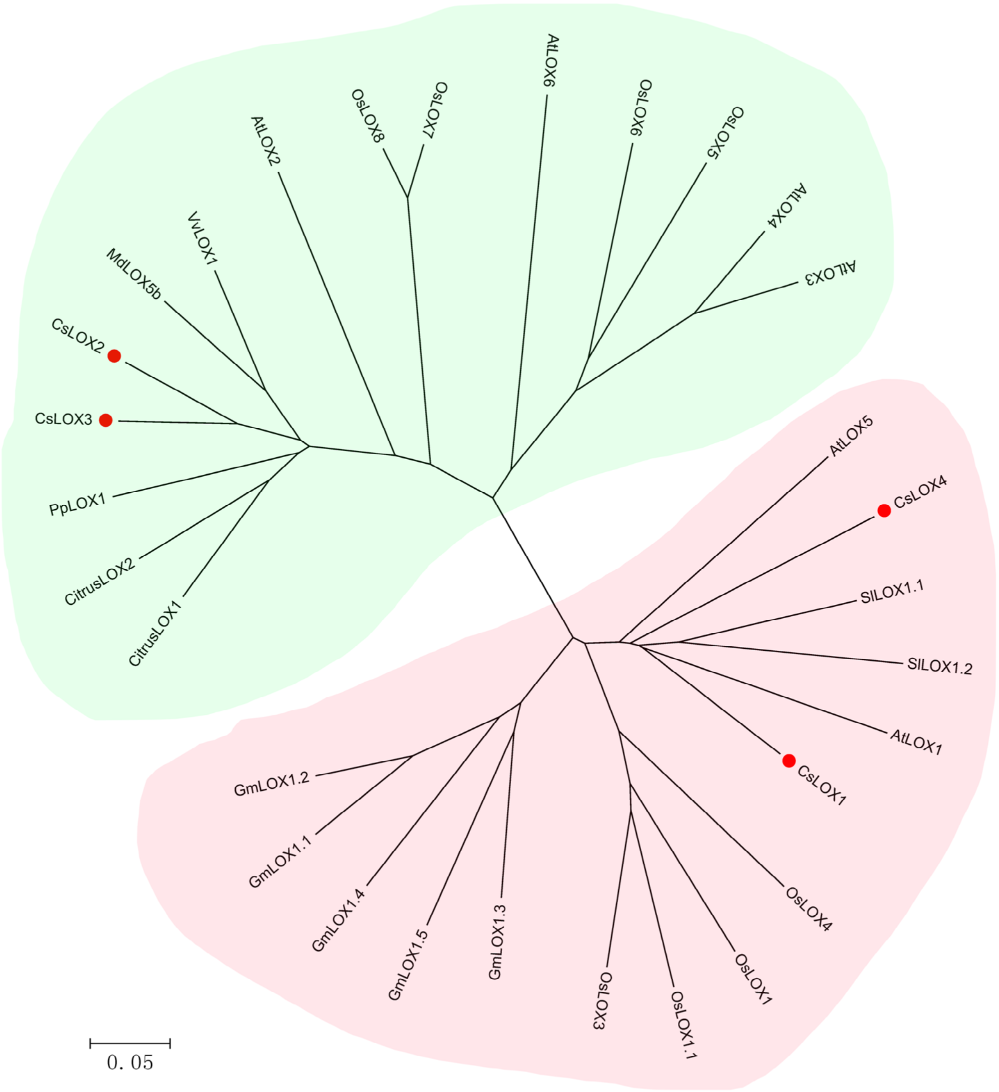

Figure S9 Phylogenetic analysis of *Camellia sinensis* (CsLOX1 , CsLOX2, CsLOX3 and CsLOX4,) and other plant Lipoxygenase genes. AtLOX1, Q06327; AtLOX2, P38418; AtLOX3, Q9LNR3; AtLOX4, Q9FNX8; AtLOX5, Q9LUW0; AtLOX6, Q9CAG3; CitrusLOX1, V4U565; CitrusLOX2, V4TFS9; GmLOX1.1, P08170; GmLOX1.2, P09439; GmLOX1.3, P09186; GmLOX1.4, P24095; GmLOX1.5, P38417; MdLOX5b, S4UL41; OsLOX1, Q76I22; OsLOX1.1, P29250; OsLOX3, Q7G794; OsLOX4, Q53RB0; OsLOX5, Q7XV13; OsLOX6, Q8H016; OsLOX7, P38419; OsLOX8, Q84YK8;PpLOX1, M5W1M2; SlLOX1.2, P38416; SlLOX1.2, P38415; VvLOX1, F6HB91. Method: The phylogenetic tree constructed using MEGA (version 5.0), and the sequence alignment was used for a Neighbor–Joining (NJ) tree using default parameters in MEGA. Bootstrap analysis of the NJ tree was performed using 1000 replicates.

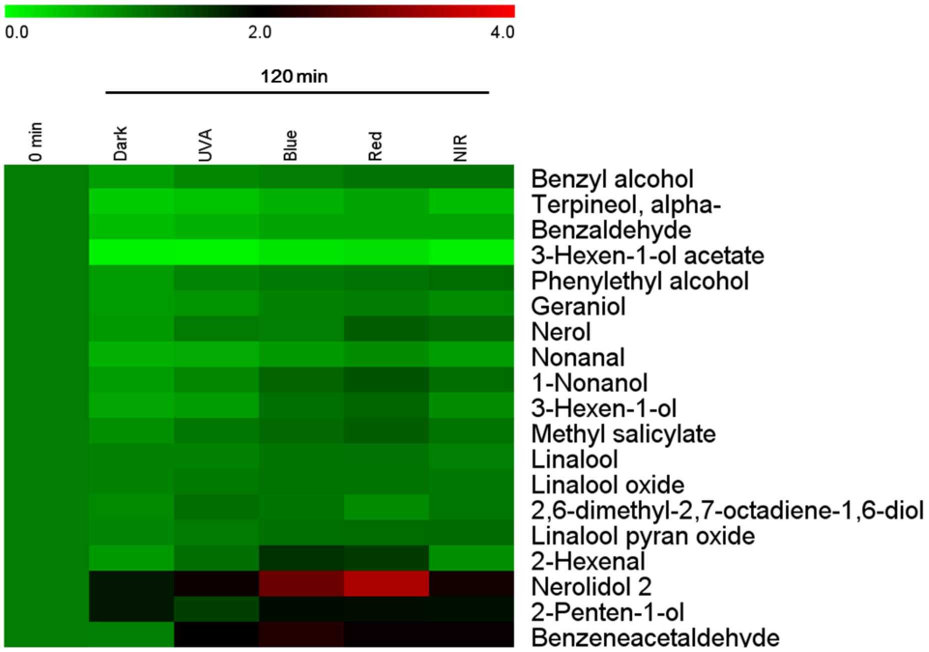

Figure S10 Comparison of volatiles in 0 min and 120 min treatments of dark, UVA, blue light, red light, and near-infrared (NIR) on postharvest tea leaves

The 0 min treatment was normalized as 1. The data were calculated based on mean values (n=3). The original data for making this figure is shown in Table S3 (Supplementary information).

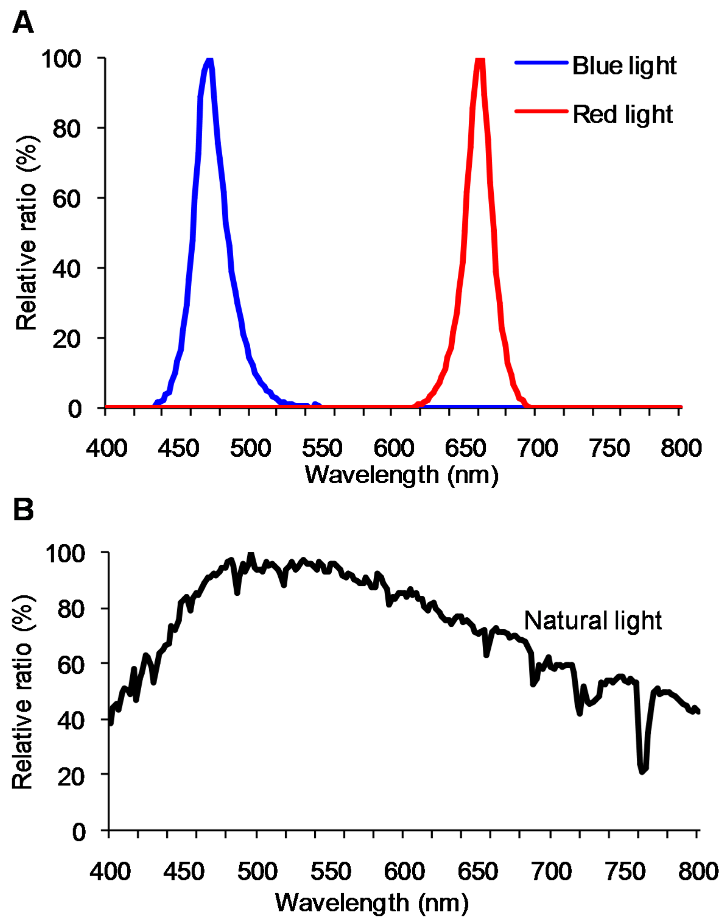

Figure S11 Light conditions under blue light and red light (A), and natural light (B)

The highest light intensity was defined as 100%.
